# The Phagocytic Code Regulating Phagocytosis of Mammalian Cells

**DOI:** 10.3389/fimmu.2021.629979

**Published:** 2021-06-09

**Authors:** Tom O. J. Cockram, Jacob M. Dundee, Alma S. Popescu, Guy C. Brown

**Affiliations:** Department of Biochemistry, University of Cambridge, Cambridge, United Kingdom

**Keywords:** phagocytosis, cell, signal, opsonin, immunity, cancer, neurodegeneration, signalling

## Abstract

Mammalian phagocytes can phagocytose (i.e. eat) other mammalian cells in the body if they display certain signals, and this phagocytosis plays fundamental roles in development, cell turnover, tissue homeostasis and disease prevention. To phagocytose the correct cells, phagocytes must discriminate which cells to eat using a ‘phagocytic code’ - a set of over 50 known phagocytic signals determining whether a cell is eaten or not - comprising find-me signals, eat-me signals, don’t-eat-me signals and opsonins. Most opsonins require binding to eat-me signals – for example, the opsonins galectin-3, calreticulin and C1q bind asialoglycan eat-me signals on target cells - to induce phagocytosis. Some proteins act as ‘self-opsonins’, while others are ‘negative opsonins’ or ‘phagocyte suppressants’, inhibiting phagocytosis. We review known phagocytic signals here, both established and novel, and how they integrate to regulate phagocytosis of several mammalian targets - including excess cells in development, senescent and aged cells, infected cells, cancer cells, dead or dying cells, cell debris and neuronal synapses. Understanding the phagocytic code, and how it goes wrong, may enable novel therapies for multiple pathologies with too much or too little phagocytosis, such as: infectious disease, cancer, neurodegeneration, psychiatric disease, cardiovascular disease, ageing and auto-immune disease.

## Introduction

Every second of a human life, about two million senescent red blood cells and one million apoptotic white blood cells are eliminated by phagocytosis, the cellular process of engulfing and degrading extracellular material (1). Countless more dead or dying host cells, pathogenic microbes, infected cells, cancer cells, excess synapses and cellular debris are similarly engulfed and digested. These phagocytic targets have to be accurately distinguished from around 30 trillion healthy cells. How is this distinction made, given that the phagocyte cannot look inside the cells it encounters, but can only read surface signals using its own surface receptors? The task may have seemed relatively simple when we only knew of two or three signals that regulated this phagocytosis, but we now know of over 50 such signals, some stimulating and some inhibiting phagocytosis. How is this information integrated to decide whether to eat or not to eat?

In this review, we outline the components of the phagocytic code i.e. established and novel find-me signals, eat-me signals, don’t eat-me signals and opsonins used to discriminate which mammalian cells (or sub-cellular material) to phagocytose. We have largely excluded signals mediating phagocytosis of pathogens, as this is a separate field well reviewed by others (2, 3), although many signals are known to regulate phagocytosis of both pathogens and mammalian cells. We describe how each eat-me signal, don’t-eat-me signal or opsonin interacts with receptors on phagocytes, as this is fundamental to operation of the phagocytic code. We then illustrate how these interactions determine the phagocytosis of particular targets, including: healthy cells, excess cells in development, senescent and aged cells, infected cells, cancer cells, dead or dying cells, cell debris and neuronal synapses. We outline how phagocytic signalling may go wrong in disease, and how this may inform novel therapies. Finally, we then offer some generalisations as to how the phagocytic code operates and integrates phagocytic signals.

*Definitions*. Terms used in the field of phagocytosis can be ambiguous, so it is important to clarify their definitions. Phagocytosis is a cellular process of engulfment and digestion of extracellular material > 0.5 microns in size, including other cells. We will call the cell doing the phagocytosis the phagocyte, and the cell to be phagocytosed the target cell. A find-me signal is a molecule released from a target cell to attract a phagocyte toward that cell. All find-me signals are chemotactic factors, but not all chemotactic factors are find-me signals, as many chemotactic factors are released by immune cells to attract other immune cells to remove pathogens and damage, but not to phagocytose the immune cells. An eat-me signal is a signal exposed on or released from a cell encouraging phagocytes to phagocytose that cell. A don’t-eat-me signal is a signal exposed on or released from a cell discouraging phagocytes to phagocytose that cell. An opsonin is a normally soluble, extracellular molecule, not derived from the phagocytosed cell, which, when bound to a cell, encourages phagocytes to phagocytose that cell. Opsonins can be confused with eat-me signals (as both stimulate phagocytosis of target cells), and indeed there is some overlap, but the fundamental distinction is that eat-me signals originate from the target cell, whereas opsonins do not. However, there are some eat-me signals released by target cells that can bind back onto the target cell to act as ‘self-opsonins’. The original opsonins were antibodies and complement proteins, but ‘opsonin’ now refers to any external molecule capable of bridging between target cells and phagocyte to stimulates phagocytosis of the target cell. The phagocytic code is the set of signals that determine whether a cell is phagocytosed by a phagocyte or not. A phagocyte is a cell capable of phagocytosing. The main professional phagocytes (cells specialised for phagocytosis) are: neutrophils, monocytes, dendritic cells and macrophages. Some tissues have specialised macrophages that enter the tissue prior to birth (e.g. microglia in the CNS); other macrophages differentiate from blood monocytes recruited into tissues during inflammation. A number of other cell types (such as fibroblasts) can act as non-professional phagocytes, capable of phagocytosing small, local targets, but with limited capacity to migrate, detect, engulf and digest targets. A target cell here means a cell potentially phagocytosed by a phagocyte. Targets include apoptotic and necrotic cells: necrotic cells have a ruptured cell membrane, whereas apoptotic cells have an intact cell membrane but exposed phosphatidylserine due to caspase activation. A phagocytic receptor is a receptor on a phagocyte that specifically regulates phagocytosis by responding to eat-me signals, don’t-eat-me signals or opsonins. A negative opsonin is a normally soluble, extracellular molecule, which, when bound to the target cell, discourages phagocytes from phagocytosing that cell. A phagocyte suppressant is a normally soluble, extracellular molecule, which, when bound to a phagocyte discourages it from phagocytosing targets. ‘Macrophage phagocytosis’ is ambiguous as it may refer to ‘phagocytosis of macrophages’ or ‘phagocytosis by macrophages’, and this can cause confusion. We will use the term to mean the latter, so in general ‘X phagocytosis’ will mean ‘phagocytosis by X’.

## The Phagocytic Code

### Find-Me Signals

**Figure 1:**
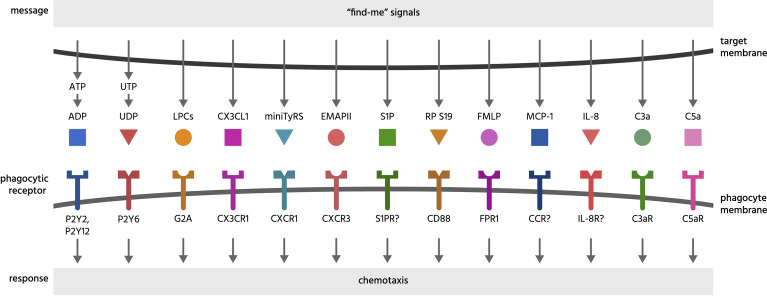
Find-me signals and their receptors. Find-me signals are molecules released from a cell to attract phagocytes, resulting in phagocytosis of that cell. The figure illustrates find-me signals released from mammalian target cells, and their putative receptors on phagocytes, mediating the recruitment of phagocytes to the target cells. ADP, adenosine-5’-diphosphate; ATP, adenosine-5’-triphosphate; C3a, complement component C3a; C5a, complement component C5a; C3aR, C3a receptor; C5aR, C5a receptor (CD88); CCR, chemokine receptor; CD88, cluster of differentiation 88 (a complement component C5a receptor); CX3CL1, chemokine C-X3-C ligand 1 (fractalkine); CX3CR1, C-X3-C chemokine receptor; CXCR1, C-X-C chemokine receptor type 1; CXCR3, C-X-C chemokine receptor type 3; EMAP II, endothelial monocyte-activating polypeptide II; FMLP, N-formylmethionyl-leucyl-phenylalanine; FPR1, formyl peptide receptor 1; G2A, G protein coupled receptor 132 (GPR132); IL-8, interleukin 8; IL-8R, interleukin 8 receptor; LPC, lysophosphatidylcholine; MCP-1, monocyte chemoattractant protein 1; miniTyRS, split tyrosyl tRNA synthetase; RP S19, (dimerised) ribosomal protein S19; S1P, sphingosine-1-phosphate; S1PR, sphingosine-1-phosphate receptor; P2Y, a family of purinergic receptors; UDP, uridine-5’-diphosphate; UTP, uridine-5’-triphosphate.

Find-me signals are molecules released from a cell to attract phagocytes, resulting in phagocytosis of that cell, and include proteins, lipids and nucleotides (4). **Table 1** includes known find-eat-me signals, whilst **Figure 1** illustrates their binding receptors.

**Table 1:** Find-me signals.

Name	Receptor	Cells involved
**Complement C3a and C5a**	C3a receptor (5)	Necrotic cells attracting neutrophils and monocytes (5–7)
	C5a receptor (6, 7)	
**Dimerised ribosomal protein S19**	C5a receptor (CD88) (8, 9)	Apoptotic cell attraction of monocytes (10, 11)
** (RP S19)**		
**Endothelial monocyte-activating polypeptide II (EMAPII)**	CXCR3 (12, 13)	Apoptotic & cancer cell attraction of neutrophils and monocytes (14, 15)
**Formyl peptides**	FPR1 (16)	Necrotic cells attracting neutrophils (16)
** (e.g. FMLP)**		
**Fractalkine (CX3CL1)**	Fracktalkine receptor	Apoptotic cells attracting macrophages or monocytes (17, 18). Synapses attracting microglia (19, 20)
	(CX3CR1) (21, 22)	
**Interleukin-8 (IL-8)**	Type 1 IL-8 receptors (23)	Fas-induced apoptosis attracting monocytes and neutrophils (24)
**Lysophosphatidycholine (LPC)**	Possibly G2A (25)	Apoptotic cells attracting macrophages or monocytes (26)
**Monocyte chemoattractant protein 1 (MCP-1, a.k.a CCL-2)**	CCR2 (27)	Fas-induced apoptosis attracting monocytes and neutrophils (24)
**Nucleotides:**	P2Y2 (28), P2Y6 (28), P2Y12 (29)	Apoptotic thymocytes & T-cells attracting monocytes, macrophages & neutrophils (29, 30),
** ATP, ADP, UTP, UDP**		
**Sphingosine-1-phosphate (S1P)**	S1P receptors 1-5	Apoptotic cells attracting phagocytes (31).
	(unclear which involved) (4)	
**Split human tyrosyl-tRNA synthetase (mini TyrRS)**	Possibly IL-8RA (32)	Apoptotic cell attraction of leukocytes & monocytes (32)

***Complement components C3a and C5a*** are protein components of the complement system, generated from C3 and C5 respectively by proteolysis once the complement system is activated. Complement can be activated on necrotic cells particularly, generating C3a and C5a, which are both chemotactic for a variety of phagocytes (33). In muscle injury, C3a was found to be necessary to recruit monocytes (via the C3a receptor) to the necrotic tissue (5). In liver injury, C5a was necessary to recruit neutrophils (6). Both C3a and C5a are involved in recruitment of neutrophils and monocytes to arthritic joints (7).

***Dimerised ribosomal protein S19 (RP S19)*** is a find-me signal for monocytes, first discovered in the context of rheumatoid arthritis (34). Later it was found to be released by apoptotic cells, and bind to the complement C5a receptor CD88 (8, 9). It is unclear how RP S19 is secreted by apoptotic cells, and may actually be released from apoptotic cells that have become necrotic (secondary necrosis).

***Endothelial monocyte-activating polypeptide II (EMAP II)*** is a chemotactic cytokine released by apoptotic and cancer cells to recruit neutrophils and monocytes (14, 15). The main receptor is thought to be CXCR_3_ (12). It is unclear how EMAP II is released from cells, and may be released during necrosis.

***Formyl peptides (including N-formylmethionyl-leucyl-phenylalanine, fMLP)*** are found in mitochondria, and are released from necrotic cells, inducing migration of neutrophils *via* formyl peptide receptor 1 (FPR1) (16).

***Fractalkine (CX_3_CL1)*** is ubiquitously expressed as a membrane-anchored protein, but can be enzymatically cleaved to a soluble form by cells undergoing apoptosis, such as lymphocytes (17) and germinal B cells (18), to induce migration of macrophages or monocytes. Chemotactic responses to fractalkine act *via* the fractalkine receptor CX_3_CR1, expressed by macrophages, natural killer cells, T cells and circulating monocytes (21, 22). In the brain, soluble fractalkine is released by the metalloprotease ADAM10 on neurons, and may drive migration of microglia to developing neurites for synaptic pruning during circuitry development (19, 20, 35, 36).

***Interleukin-8 (IL-8) and monocyte chemoattractant protein 1 (MCP-1)*** can be released from multiple cell types when apoptosis is induced by Fas, and chemoattract monocytes and neutrophils (24). It is not clear whether this is specific to Fas-induced apoptosis.

***Lysophosphatidylcholine*** is a soluble lipid generated from membrane phosphatidylcholine by the action of phospholipase A_2_ (37). Chemotaxis may be induced *via* the receptor G2A (25), although lysophosphatidylcholine may also block this receptor in certain contexts (38). Lysophosphatidylcholine can be released from apoptotic cells following caspase-3 mediated activation of phospholipase A_2_ to attract monocytes and macrophages (26).

***Nucleotides: adeonise-5’-triphosphate (ATP), adeonise-5’-diphosphate (ADP), uridine-5’-triphosphate (UTP) and uridine-5’-diphosphate (UDP)*** are released from a variety of cells undergoing apoptosis, including thymocytes and T-cells (39), and these extracellular nucleotides can promote migration of monocytes, macrophages and neutrophils to apoptotic cells in vitro and in vivo (29, 30). Nucleotide release from cells with intact membranes occurs via connexin or pannexin channels, and release from apoptotic cells can result from caspase-dependent cleavage of pannexins (40, 41). Phagocytes express a range of ionotropic P2X and metabotropic P2Y receptors for nucleotides that may mediate chemotaxis (42). In particular, the UTP and ATP-sensing P2Y_2_ receptor mediates macrophage chemotaxis towards apoptotic T cells and thymocytes (39). The ATP and ADP-sensing P2Y_12_ receptor mediates microglial migration and processes extension toward sites of brain damage in vivo (29). UDP induces migration of immature dendritic cells via the P2Y_6_ receptor (28). UDP may also chemoattract neutrophils, eosinophils and natural killer cells (43). Extracellular ectonucleotidases can degrade ATP/ADP and UTP/UDP to adenosine and uridine respectively and so may prevent these nucleotides from acting as find-me signals (44). For example, Thompson et al. showed that knockout of the ectonucleotidase CD73 increased lymphocyte migration to draining lymph nodes (45). Degradation of UDP by nucleotidases may also prevent it from acting as an eat-me signal (see ‘eat-me signals’ section below), and degradation of ATP/ADP generates adenosine, which may act as a don’t eat-me signal (see ‘don’t-eat-me’ section below).

***Sphingosine-1-phosphate (S1P)*** is also released during apoptosis to induce phagocyte migration (31). S1P can activate five different S1P receptors S1P_1-5_, but which mediates chemotaxis is unclear (4).

***Split tyrosyl tRNA synthetase (mini TyrRS*)** acts as a find-me signal for apoptotic cells once cleaved by the extracellular protease elastase (32). Cleavage produces two fragments, both acting as chemoattractants. The N-terminal fragment binds to the interleukin-8 type A receptor (32), although this has not been shown to mediate chemoattraction.

### Eat-Me Signals

**Figure 2:**
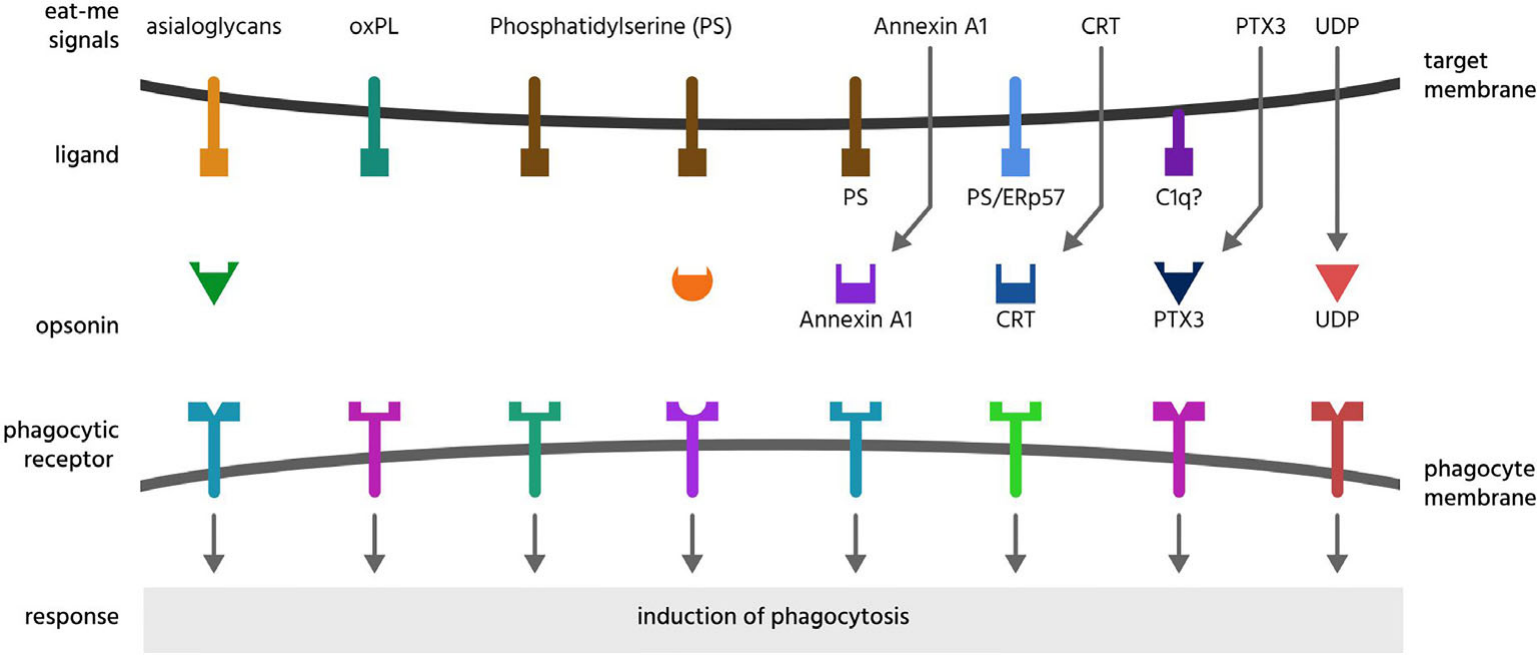
Eat-me signals. Eat-me signals are molecules exposed on or released from a target cell to directly induce phagocytosis of that cell by a phagocyte. The figure illustrates eat-me signals on or from mammalian target cells. Asialoglycans are desialylated glycoproteins or glycolipids, that can bind multiple opsonins, and induce phagocytosis *via* multiple receptors. Oxidised phospholipids (oxPL) can induce phagocytosis *via* receptors CD36 and LOX-1. Phosphatidylserine (PS) can induce phagocytosis either directly *via* multiple phagocytic receptors or indirectly *via* binding multiple opsonins. Annexin A1, CRT (calreticulin) and PTX3 (pentraxin 3) are soluble proteins released onto the surface of target cells, where they bind ligands: PS (phosphatidylserine), C1q (complement component C1q) or ERp57 (endoplasmic reticulum resident protein p57). UDP (uridine-5’-diphosphate) can induce phagocytosis *via* activating the P2Y6 receptor.

Eat-me signals are molecules exposed on or released from a target cell to directly induce phagocytosis by a phagocyte. Most eat-me signals (such as phosphatidylserine) are anchored in the target cell membrane, but some (such as calreticulin) are soluble proteins bound to the cell surface, and may be released and bind back onto the target cell. These overlap somewhat with opsonins and could be regarded as ‘self-opsonins’. Below we discuss all potential eat-me signals, but only those with reasonable evidence are listed in **Table 2** and illustrated in **Figure 2** with their binding partners.

**Table 2:** Eat-me signals.

Name	Opsonin(s)	Phagocytic receptor(s)	Cells and targets involved
**Annexin A1 (AnxA1/ANX1)**	Self-opsonin	PSR (46)	Endothelial cell phagocytosis of apoptotic T cells (46)
**Asialoglycans**	Calreticulin (47), C1q (48), galectin-3 (49), MBL (50)	CR3 (for N-acetyl-glucosamine residues (51), MGL & AMR (for galactose residues (52)	Macrophage phagocytosis of ageing neutrophils (47), neuroendocrine cells (49), synapses (48, 53) and cell debris (49); phagocytosis of apoptotic lymphoblasts by monocyte-derived phagocytes (54)
**Calreticulin**	C1q (55–57)	LRP1 (58, 59)	Macrophage phagocytosis of viable and apoptotic leukocytes and erythrocytes (58)
	Self-opsonin		
**Oxidised phospholipids**	None, but may vary with phospholipid and oxidation	CD36 (60–62), possibly LOX-1 (63, 64), TLR2 (65)	Macrophage phagocytosis of apoptotic thymocytes (66) and apoptotic HL60 cells (60), retinal pigment epithelial cell phagocytosis of photoreceptor outer segments (62)
**Pentraxin-3**	Self-opsonin	Unknown	Macrophage phagocytosis of apoptotic neutrophils (67) and apoptotic macrophages (68)
**Phosphatidylserine**	Annexin A1 (46, 69), ApoH (β2-GP1) (70), calreticulin (56, 71), CCN1 (72), Gas6 (73–75), MFG-E8 (76–79), protein S (80, 81), C1q (56, 82), C3b & iC3b (83), MBL (84), SP-A (85), TSP-1 (86)	BAI-1 (87), CD300f (CLM-1) (88), CD36 (89, 90), LOX-1 (91), PSR (92, 93), RAGE (94), Stabilin-1 (95), Stabilin-2 (96, 97), TIM-1 (98), TIM-4 (98–102), TREM2 (82, 103, 104)	Macrophage phagocytosis of synapses (56), apoptotic thymocytes (87, 88, 94, 98, 105), apoptotic neutrophils (90, 94), apoptotic T cells (93), aged red blood cells (95), apoptotic neurons (104), viable neurons (97,168,173,; epithelial cell phagocytosis of apoptotic T cells (93); fibroblast phagocytosis of apoptotic T cells (92, 93), monocytes (98), eryptotic red blood cells (98), glial phagocytosis of axonal debris (106)
**Uridine-5’-diphosphate**	None	P2Y6 (107, 108)	Microglial phagocytosis of neurons during stress or inflammation (107, 108)

***Annexin A1 (AnxA1)*** is a soluble protein known to facilitate a return to homeostasis following inflammation (109). AnxA1 can be externalised by apoptotic cells to induce phagocytosis of them by endothelial cells, possibly *via* the phosphatidylserine receptor (46). AnxA1 can bind phosphatidylserine, and colocalises with phosphatidylserine on apoptotic cells. Apoptotic lymphocytes may also expose AnxA1, inducing phagocytosis by macrophages (110). AnxA1 can also act as an opsonin by binding phosphatidylserine on target cells (see below). Thus, AnxA1 could be regarded as a self-opsonin.

**Figure 3:**
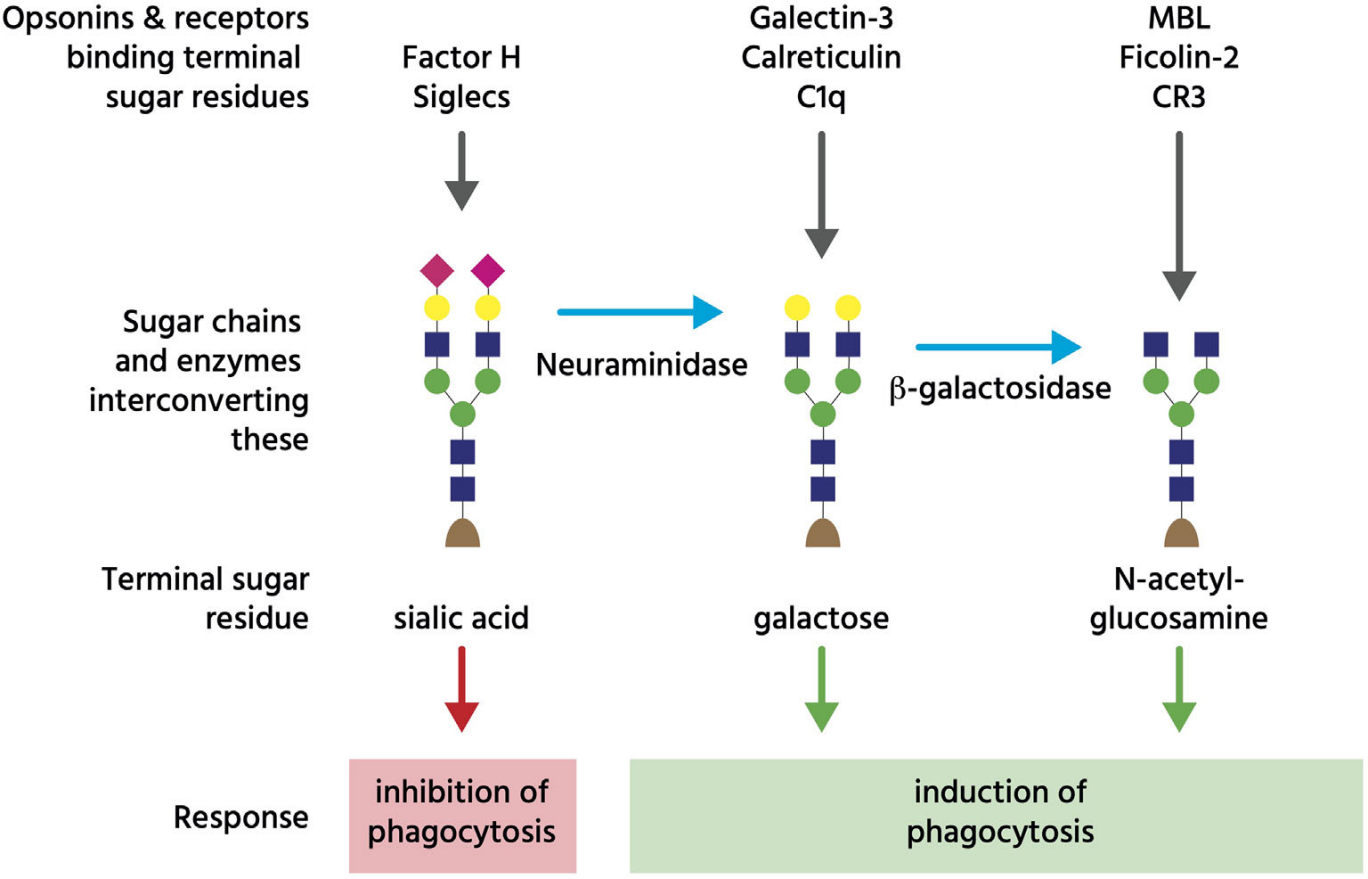
Glycans regulating phagocytosis. The coloured shapes illustrate sugar residues in a typical glycan chain on an N-glycosylated cell-surface glycoprotein, where the terminal (monosaccharide) sugar is normally sialic acid (pink diamond), which can bind either Factor H to inhibit complement, or Siglec receptors to inhibit phagocytosis. However, neuraminidases can remove that terminal sialic acid residue to reveal galactose residues (yellow circle), which bind opsonins galectin-3, calreticulin and C1q. Subsequently, beta-galactosidase can remove terminal galactose residues to reveal N-acetyl-glucosamine residues (blue square), which bind opsonins (and complement regulators) MBL (mannan binding lectin) and ficolin-2, as well as complement receptor 3 (CR3). This figure is a considerable simplification of actual sugar chains and their regulation of phagocytosis.

***Asialoglycans*** are glycans (molecules with sugar chains) that lack sialic acid, a monosaccharide normally at the end of glycan chains on glycoproteins and gangliosides (**Figure 3**). Desialylation (removal of sialic acid residues) of glycans can occur *via* neuraminidases (111), which can translocate to the cell surface during inflammation (112), thus generating surface asialoglycans. Such asialogylcans can then act as eat-me signals, for example for apoptotic cells (54), where the phagocytic signal of asialogylcans is relayed by binding opsonins. For example, the opsonin galectin-3 binds cell-surface galactose residues normally hidden by sialic acid, so galectin-3 binding to neuroendocrine cells was increased by desialylation, resulting in their phagocytosis by microglia (brain-resident macrophages) (49). Similarly, the binding of calreticulin to galactose-containing Galβ1→4GlcNAc residues exposed by desialylated neutrophils induced their phagocytosis by macrophages (47). Furthermore, the binding of the complement component C1q to neurites was greatly increased by desialylation, resulting in phagocytosis of C1q-opsonised neurites by microglia (48), possibly *via* C1q binding to exposed galactose (113). Exposed galactose residues on aged platelets induced their phagocytosis by liver macrophages *via* the macrophage galactose lectin (MGL) and Ashwell-Morell receptor (AMR), both of which bind exposed galactose residues directly (52). Desialylation exposes galactose residues, but removal of further sugar residues (e.g. by β-galactosidase and β-hexosaminidase) exposes N-acetylglucosamine and mannose residues, which are bound by mannose-binding lectin (MBL) (50). Exposed N-acetylglucosamine on aged platelets also directly activated phagocytosis *via* the lectin domain of the phagocytic complement receptor CR3 (51). Thus, asialoglycans act as eat-me signals in diverse tissues and cell-types (**Figure 3**).

***Calreticulin*** is a lectin (sugar-binding protein) that normally resides in the endoplasmic reticulum (ER), but can be translocated to the surface of the cell during apoptosis or ER stress (58, 114). Surface-exposed calreticulin acts as an eat-me signal to phagocytes *via* the LRP1 receptor, which mediates macrophage phagocytosis of healthy or apoptotic leukocytes, erythrocytes, and neurons (58, 114, 115). Calreticulin can bind directly to LRP1 (116), but it may also promote phagocytosis by binding the opsonin C1q (55), which can itself activate LRP1 to induce phagocytosis (117). Calreticulin can bind phosphatidylserine (56), and so may signal to phagocytes either alone or in association with exposed phosphatidylserine. Phagocytic signalling by apoptotic or ER-stressed cancer cells may also occur *via* translocation to the cell surface of calreticulin bound to ERp57 (114). However, as calreticulin is soluble and binding to ERp47 and phosphatidylserine is not covalent, calreticulin may also be released from cells. Indeed, calreticulin can be secreted as a soluble protein and act as an opsonin by binding asialoglycans on target cells (see *‘Asialoglycan’* section below), and potentially by binding phosphatidylserine or ERp57. Thus, calreticulin could be regarded as a ‘self-opsonin’ i.e. something released from and binding back onto a target cell to stimulate phagocytosis of the cell.

***Deoxyribonucleic acid (DNA)*** - like histones, genomic DNA translocates from the nucleus to the cell-surface during necrosis and apoptosis, where it may act as an eat-me signal by binding opsonins. Xu et al. (118) found that the opsonin properdin strongly binds both double-stranded and single-stranded DNA, and associates with DNA on the surface of both necrotic and apoptotic cells. Jensen et al. (119) showed Ficolin-2 binds DNA, and induces phagocytosis of necrotic (but not apoptotic) T cells by monocytes. Additionally, C1q and C3 both directly bind DNA, and their recruitment at the surface of apoptotic cells is impaired by enzymatic degradation of DNA (120). Thus, DNA might act as an eat-me signal for cells exposing DNA, although it has not been shown that degrading cell-surface DNA inhibits phagocytosis of such cells. DNA does not itself integrate into the cell membrane, so may be better described as a self-opsonin or complement activator, although what DNA binds on the surface of dying cells is unclear.

***Histones*** are normally located within the nucleus of all mammalian cells, but can appear on the cell surface during apoptosis (121). Here, they may act as eat-me signals by binding the opsonin apoJ (clusterin), thus facilitating phagocytic clearance of the apoptotic cell by macrophages (122). However, it is not yet known whether blocking histones at the surface of a target cell can inhibit phagocytosis.

***Intercellular adhesion molecule 3 (ICAM-3, CD50)*** is constitutively expressed by leukocytes and mediates cell-cell adhesion by interacting with specific integrin receptors (61). ICAM-3 has been called an eat-me signal because antibodies blocking ICAM-3 on apoptotic neutrophils inhibit macrophage phagocytosis of the neutrophils in culture (123). ICAM-3 can directly bind the integrin receptor LFA-1 (lymphocyte function-associated antigen 1) (124), and knocking down or blocking LFA-1 in macrophages inhibited their phagocytosis of apoptotic neutrophils (123). Phagocytosis of ICAM-3-expressing apoptotic B cells, T cells and neutrophils may also involve the CD14 receptor on macrophages (125). As LFA-1 and CD14 are not phagocytic receptors, it seems likely that the role of ICAM-3 in phagocytosis is primarily adhesive, whereas phagocytosis *per se* is triggered by other signals (126). That ICAM-3 is not itself an eat-me signal is supported by the finding that ICAM-3 does not appear to change form and has somewhat lower expression during apoptosis (125). However, apoptotic leukocytes can release ICAM-3 as microparticles, which can chemoattract macrophages and may act as find-me signals (126).

***Oxidized phospholipids (oxPL)***. Phospholipids can be oxidised by a variety of processes, and some oxidised phospholipids can act as eat-me signals. Monoclonal antibodies that target oxidation-specific epitopes on surface phospholipids have been shown to bind apoptotic cells and block their phagocytosis by macrophages, whilst the same antibodies failed to bind non-apoptotic cells (66). Interestingly, Greenberg et al. (60) found that incorporating oxidised (but not non-oxidised) phosphatidylserine into healthy cells was sufficient to induce their phagocytosis by macrophages. Thus, in certain contexts, phosphatidylserine may be insufficient to induce phagocytosis without oxidation. The scavenger receptor CD36 can directly bind oxPL (61), and mediate macrophage phagocytosis of apoptotic cells in culture (60). CD36 recognition of oxPL also mediated phagocytosis of photoreceptor outer segments by retinal pigment epithelial (62). The lectin-like OxLDL receptor 1 (LOX-1) can mediate phagocytosis of aged and apoptotic cells, possibly by binding oxidised low-density lipoproteins (LDLs) on these cells (63). Oxidized phosphatidylethanolamine exposed on the surface of cells undergoing ferroptosis (an iron-dependent form of programmed cell death) triggers macrophage phagocytosis of these cells *via* the macrophage toll-like receptor 2 (TLR2) (65).

***Pentraxin-3 (PTX3)*** is a conserved member of the pentraxin family of acute phase proteins, and can translocate from intracellular granules to the surface of neutrophils during apoptosis, thereby promoting their phagocytosis by macrophages (67). PTX3 on the surface of apoptotic macrophages can induce phagocytosis of these apoptotic cells by non-apoptotic macrophages (68), although in this case it is unclear whether the surface PTX3 originated from inside or outside the apoptotic cell. PTX3 is a soluble protein that can also function as an opsonin (see below). Thus, PTX3 could be regarded as a self-opsonin for some apoptotic cells.

***Phosphatidylserine*** is the most widely-documented eat-me signal. Phosphatidylserine constitutes approximately 10% of plasma membrane phospholipids, but in healthy cells is contained within the inner leaflet of the membrane *via* ATP-powered aminophospholipid translocases (127). During apoptosis, surface exposure of phosphatidylserine may increase over 100-fold within 1 or 2 hours (128) due to decreased translocase activity and increased activity of phospholipid scramblases (proteins that promote phosphatidylserine externalisation to the outer surface), where it acts as an eat-me signal. Blocking exposed phosphatidylserine on apoptotic cells by adding the phosphatidylserine-binding protein annexin V can fully prevent phagocytosis by macrophages (129).

There are several different phagocytic receptors for phosphatidylserine, which either bind to phosphatidylserine directly, or indirectly *via* opsonins. Non-opsonic receptors for phosphatidylserine include T-cell immunoglobulin and mucin (TIM) family receptors TIM-1, TIM-3 and TIM-4 (98, 99), brain angiogenesis inhibitor 1 (BAI-1) (87) and stabilin-2 (96) (**Table 2**). Opsonins binding phosphatidylserine (and their corresponding phagocytic receptors) include: Annexin A1 *via* formyl peptide receptor 2 (FPR2) or the phosphatidylserine receptor (PSR) (46, 69), apolipoprotein H *via* an unconfirmed receptor (70), calreticulin *via* lipoprotein receptor-related protein 1 (LRP1) (56, 71), milk fat globule-epidermal growth factor E8 (MFG-E8) *via* the integrin receptor α_v_β_3_ (a vitronectin receptor, or VNR) (76), Cellular Communication Network Factor 1 (CCN1) *via* the integrin receptors α_v_β_3_ and α_v_β_5_ (72), and growth arrest-specific 6 (Gas6) & protein S *via* TAM receptors (Tyro, Axl and MerTK) (73–75, 80, 81) (**Table 2**).

Phosphatidylserine exposure was considered to be exclusively an apoptotic marker. However, transient phosphatidylserine exposure by live cells (not committed to death) has also been reported (130), and such exposure can be sufficient to induce death of these cells directly *via* ‘phagoptosis’ (death by phagocytosis) (131). Phosphatidylserine exposure on sub-cellular targets such as neuronal synapses (82) and axonal debris (106) has also been reported to facilitate their phagocytic clearance, which may be relevant in brain development and pathology.

Interestingly, phosphatidylserine can also negatively regulate phagocytosis by activating the inhibitory receptor CD300a on macrophages to inhibit phagocytosis (88). The function of this dual signalling is unclear, but would enable particular phagocytes to downregulate phagocytosis of phosphatidylserine-exposed cells when CD300a is upregulated.

***Signalling lymphocytic activation molecule F7 (SLAMF7)*** is constitutively expressed on haematopoietic cells, and mediates macrophage phagocytosis of haematopoietic cells when CD47 (a don’t-eat-me signal) is blocked (132). This phagocytosis is apparently mediated by SLAM7 on target cells binding SLAM7 on macrophages, activating phagocytosis *via* CR3. Thus, SLAM7 could be regarded as a very specialised eat-me signal. However, as SLAM7 is constitutively expressed and does not appear to change as a result of CD47 blockade, it may be confusing to call it an eat-me signal.

***Uridine-5’-diphosphate (UDP)*** is an exceptional eat-me signal, as unlike classical eat-me signals, it is a nucleotide released locally from target cells to induce phagocytosis by proximal phagocytes. UDP is released from dying or stressed neurons, and activates the phagocytic P2Y6 receptor expressed by microglia to induce phagocytosis (107, 108), so may be especially important in brain homeostasis. UDP may also be a find-me signal (see previous section), but it stimulates engulfment itself several fold (107). Indeed, UDP may be better thought of as an engulfment signal than as an eat-me signal.

### Opsonins

**Figure 4:**
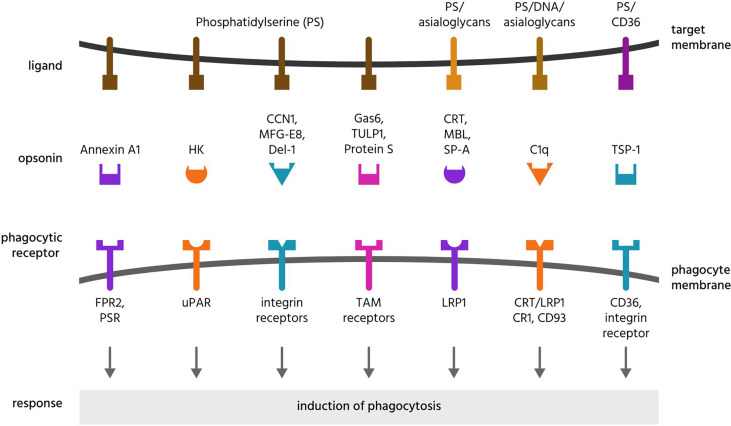
Opsonins and their ligands and receptors. Opsonins are normally soluble, extracellular proteins, which when bound to target cells induce phagocytes to phagocytose these cells. To achieve this, most opsonins bind eat-me signals on the target cell and phagocytic receptors on the phagocyte, and these are illustrated here for a variety of opsonins that bind phosphatidylserine or asialoglycans. CCN1, cellular communication network factor 1; CD, cluster of differentiation; CR1, complement receptor 1; CRT, calreticulin; Del-1, developmental endothelial locus 1; DNA, deoxyribonucleic acid; FPR2, formyl peptide receptor 2; HK, high molecular weight kininogen; LRP1, lipoprotein receptor-related protein 1; MBL, mannose-binding lectin; MFG-E8, milk fat globule-epidermal growth factor E8; PS, phosphatidylserine; PSR, phosphatidylserine receptor; SP-A, surfactant protein A; TAM receptors, Tyro, Axl and MerTK receptors; TSP-1, thrombospondin 1; TULP1, tubby-like protein 1; uPAR, urokinase plasminogen activator receptor.

**Figure 5:**
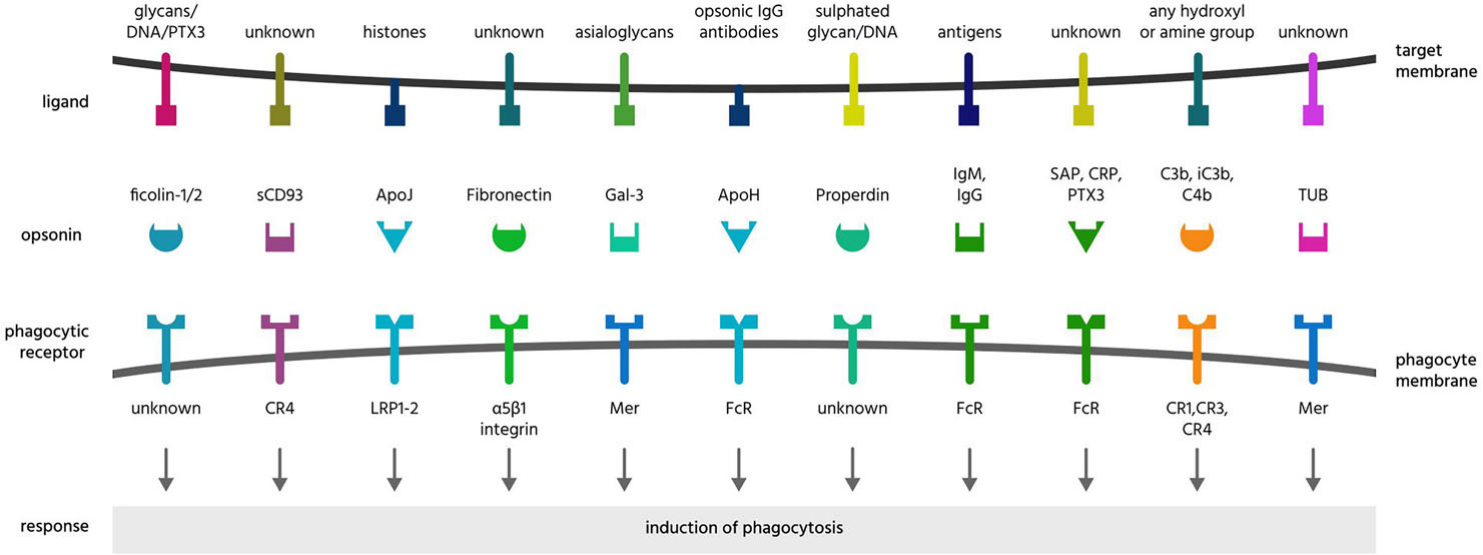
Further opsonins and their ligands and receptors. Apo, apolipoprotein; CR, complement receptor; CRP, C-reactive protein; DNA, deoxyribonucleic acid; Gal-3, galectin-3; Ig, immunoglobulin; LRP, lipoprotein receptor-related protein; PTX3, pentraxin 3; SAP, serum amyloid protein; sCD39, soluble cluster of differentiation 93; TUB, tubby.

Sometimes confused with eat-me signals, opsonins are normally soluble, extracellular proteins, which when bound to target cells induce phagocytes to phagocytose these cells (133, 134). To achieve this, opsonins must bind to both something on the target and to a phagocytic receptor on a phagocyte. Thus, opsonins are bridging proteins. As outlined above, opsonins differ fundamentally from eat-me signals in that they do not originate from the target cell. Opsonins of mammalian cells or sub-cellular targets are here reviewed, and summarised in **Table 3** and **Figures 4**, **5**.

**Table 3:** Opsonins for phagocytosis of mammalian cells/sub-cellular targets.

Name	Ligand(s) on target	Receptor(s) on phagocyte	Phagocytic function(s)
**Annexin A1 (AnxA1/ANX1)**	Phosphatidylserine (46, 69)	FPR2 (69, 135), PSR (46)	Endothelial (46) and macrophage (110, 136), phagocytosis of apoptotic T cells; macrophage phagocytosis of monocytes (135), apoptotic macrophages (136) and apoptotic neurons (69).
**Apolipoprotein E (Apo E)**	Possibly phosphatidylserine (137)	TREM2 (138), possibly LRP1 (139)	Macrophage phagocytosis of apoptotic thymocytes (140) and apoptotic neuroblastoma cells (138); astrocyte phagocytosis of synapses (141)
**Apolipoprotein J (Apo J/clusterin)**	Histones (122)	LRP1 and LRP2 (megalin) (142)	Macrophage phagocytosis of apoptotic neutrophils (122); fibroblast and epithelial cell phagocytosis of cell debris (142)
**Apolipoprotein H (β2 glycoprotein 1/β2-GP1)**	Phosphatidylserine (70, 143)	Opsonic IgG antibodies (70, 144)	Macrophage phagocytosis of apoptotic thymocytes and red blood cells (70), and platelet microvesicles (143)
**Calreticulin (CRT)**	Galactose (Galβ1➔4GlcNAc) residues of sialoglycans (47), phosphatidylserine (56, 71)	LRP1 (58)	Macrophage phagocytosis of apoptotic embryonic fibroblasts (71), ageing neutrophils (47), and viable and apoptotic leukocytes and erythrocytes (58)
**CD93, soluble form (sCD93)**	Unknown (possibly C1q (145)	CR4 (146)	Macrophage phagocytosis of apoptotic T cells (146)
**Cellular Communication Network factor 1 (CCN1, CYR61)**	Phosphatidylserine (72)	αvβ3 and αvβ5 (72)	Macrophage phagocytosis of apoptotic neutrophils (72)
**Complement C1q**	Phosphatidylserine (56, 147), calreticulin (55), galactose & N-acetyl-glucosamine residues of asialoglycans (48, 53), DNA (120)	Calreticulin/LRP1 (148), CR1 (149), CD93 (145)	Monocyte phagocytosis of apoptotic HeLa cells (55), monocyte (150) and macrophage (148, 151) phagocytosis of apoptotic T cells, macrophage phagocytosis of apoptotic erythrocytes (148), apoptotic thymocytes (152), apoptotic neurons (153), necrotic lymphocytes (154) and synapses (48, 53, 82, 155, 156).
**Complement C3b & iC3b**	Covalent reaction with exposed hydroxyl/amine groups (157)	CR1 (83), CR3 (83, 158), CR4 (83, 158)	Macrophage phagocytosis of apoptotic T cells (158), synapses (155) and myelin (159)
**Complement C3d**	Unknown	CR3 (160)	Monocyte phagocytosis of erythrocytes (160, 161)
**Complement C4 & C4b**	Non-specific binding to exposed hydroxyl/amine groups (162)	CR1 (163)	Macrophage phagocytosis of apoptotic T cells (164), apoptotic thymocytes (165) and synapses (166)
**C-reactive protein (CRP)**	Unknown (possibly C1q (167)	FcγRI (18, 168)	Macrophage phagocytosis of apoptotic T cells (169, 170), PBMC phagocytosis of erythrocytes (168)
**Developmental endothelial locus-1 (Del-1)**	Phosphatidylserine (171)	αvβ3 (171)	Macrophage phagocytosis of apoptotic neutrophils (171)
**Fibronectin**	Unknown	Possibly Fc receptors (172) or LOX-1 (173), α_5_β_1_ (174)	Monocyte phagocytosis of erythrocytes (172, 175), macrophage phagocytosis of apoptotic T cells (174)
**Ficolins**	Glycans, DNA (119) and PTX3 (176)	Unknown	Macrophage phagocytosis of necrotic (119) and apoptotic (176) T cells
**Galectin-3 (Gal-3)**	Galactose residues of asialoglycans (49)	MerTK (49, 177)	Macrophage phagocytosis of apoptotic neutrophils (178), apoptotic T cells (177), neuroendocrine cells (49) and cell debris (49); retinal pigment epithelial cell phagocytosis of neuroblastoma-derived membrane vesicles (177)
**Growth arrest-specific factor 6 (Gas6)**	Phosphatidylserine (73–75)	Axl (75, 179, 180), Tyro3 (75, 179), MerTK (75, 179, 180)	Macrophage phagocytosis of apoptotic T cells (181), apoptotic thymocytes (74, 180), retinal pigment epithelial cell phagocytosis of photoreceptor cells (182)
**High-molecular weight kininogen (HK)**	Phosphatidylserine (183)	uPAR (183)	Macrophage phagocytosis of apoptotic cells (183)
**Immunoglobulin G (IgG)**	Antigens, apo H (70, 144), FcγRIIA (184)	Fc receptors (185)	Macrophage phagocytosis of apoptotic thymocytes and red blood cells (70), apoptotic neutrophils (184) and malignant B cells (185), dendritic cell phagocytosis of apoptotic RMA cells (144)
**Immunoglobulin M (IgM)**	Lysophosphatidylcholine (186), Phosphatidylserine (187)	Fc receptors (188)	Macrophage phagocytosis of apoptotic T cells (187) and red blood cells (189)
**Mannan-binding lectin (MBL)**	Phosphatidylserine (84), N‐acetyl‐glucosamine or mannose residues of asialoglycans (50)	Calreticulin/LRP1 (148, 190)	Macrophage phagocytosis of apoptotic erythrocytes (148), apoptotic T cells (148, 191), apoptotic adipocytes (192) and apoptotic neutrophils (122); stromal vascular cell phagocytosis of apoptotic adipocytes (192)
**Milk fat globule-EGF factor 8 protein (MFG-E8/lactadherin)**	Phosphatidylserine (76–79, 128)	αvβ3 (76, 78, 79, 193), αvβ5 (193)	Macrophage (79, 194) and αvβ3-transfected fibroblast (76) phagocytosis of apoptotic thymocytes, macrophage phagocytosis of apoptotic lymphocytes (195), red blood cells (196) and neurons (197, 198), retinal pigment epithelial cell phagocytosis of photoreceptor cells (193)
**Pentraxin-3 (PTX3)**	C1q (167), ficolin-1 (176)	Unknown	Macrophage phagocytosis of apoptotic neutrophils (67), apoptotic macrophages (68) and apoptotic T cells (176).
**Properdin**	Sulphated glycosaminoglycan chains (199), DNA (118)	Unknown	Macrophage and dendritic cell phagocytosis of apoptotic T cells (199)
**Protein S (Pros1/Prot S)**	Phosphatidylserine (80, 81)	MerTK (75, 200, 201), Tyro3 (75)	Macrophage phagocytosis of apoptotic lymphoma cells (80, 202), apoptotic neutrophils (200, 203), retinal pigment epithelial cell phagocytosis of photoreceptor cells (75)
**Serum-amyloid P component (SAP)**	Phosphatidylcholine (204), C1q (205)	FcγRI (206), FcγRIIA (206, 207), FcγRIII (170, 206), FcαRI (208)	Macrophage phagocytosis of apoptotic neutrophils and apoptotic T cells (170)
**Surfactant protein A (SP-A)**	Phosphatidylserine (85), phosphatidylcholine (209)	Calreticulin/CD91 (151, 190, 210)	Macrophage phagocytosis of apoptotic neutrophils (151, 211), apoptotic T cells (151), and erythrocytes (151)
**Surfactant protein D (SP-D)**	Unknown glycan (maltose-inhibitable) (85)	Calreticulin/CD91 (151, 210)	Macrophage phagocytosis of apoptotic neutrophils (151, 211), apoptotic T cells (151), and erythrocytes (151)
**Thrombospondin (TPS-1)**	CD36 (86), phosphatidylserine (212)	CD36 (86), αvβ3 (via IAP) (86, 212–214)	Macrophage phagocytosis of apoptotic neutrophils (213), apoptotic eosinophils (214) and apoptotic fibroblasts (86)
**Tubby (TUB)**	Unknown	MerTK (215)	Retinal pigment epithelial cell phagocytosis of neuroblastoma-derived membrane vesicles and photoreceptor outer vesicles (215, 216)
**Tubby-like protein 1 (TULP1)**	Phosphatidylserine (217)	MerTK (215), possibly Tyro3 and Axl (215)	Retinal pigment epithelial cell phagocytosis of neuroblastoma-derived membrane vesicles and photoreceptor outer vesicles (215, 216), macrophage phagocytosis of apoptotic T cells and neuroblastoma-derived membrane vesicles (218)

***Annexin A1 (AnxA1)*** can act as an eat-me signal (see above) in cells undergoing apoptosis, as intracellular AnxA1 translocates to the cell surface to induce phagocytosis by phagocytes (46). However, AnxA1 is a soluble protein, and soluble AnxA1 can opsonise various cells for phagocytosis: including apoptotic T cells and macrophages (136), apoptotic neurons (69), and apoptotic monocytes (135). AnxA1 binds phosphatidylserine (219), and co-localises with phosphatidylserine on the apoptotic cell surface, suggesting that AnxA1 opsonises by binding phosphatidylserine on cells. Phagocytic receptors for AnxA1 include the formyl peptide receptor 2 (FPR2) (69, 135) and the phosphatidylserine receptor (PSR) (46).

***Antibodies*** opsonise cells by binding antigens on the target, and phagocytic Fcγ receptors on phagocytes (220). The major Fcγ receptors that promote phagocytosis of antibody-coated targets are FcγRI, FcγRIIA and FcγRIII (221). Some IgGs (immunoglobulin G) can preferentially bind apoptotic over non-apoptotic cells (144, 184). For example, antibodies to apolipoprotein H (a phosphatidylserine-binding opsonin) enhanced phagocytosis of phosphatidylserine-exposing apoptotic thymocytes (70). IgG has also been shown to opsonise apoptotic neutrophils for phagocytosis by macrophages (184), and apoptotic lymphoma cells for phagocytosis by dendritic cells (144). We have shown that antibodies targeting multiple antigens on live, malignant human B-cells enable their phagocytosis by macrophages (185). IgM antibodies binding to phosphatidylserine can also opsonise apoptotic T cells for phagocytosis by macrophages (187).

***Apolipoproteins E, J (clusterin) and H*.** Apolipoproteins are a family of lipid-binding proteins, with multiple members now recognised as opsonins. ApoE opsonises neuroblastoma cells for phagocytosis by macrophages *via* the phagocytic receptor TREM2, to which ApoE directly binds (138). ApoE has also been shown to opsonise apoptotic thymocytes for phagocytosis by peripheral macrophages (140), and can enhance phagocytosis of neuronal synapses by astrocytes (141). ApoE may bind phosphatidylserine to mediate its opsonic effects (137), although such binding is unconfirmed. ApoE can also bind and activate the phagocytic receptor LRP1 (139), which could also relay the phagocytic signal of ApoE, but again this is unconfirmed. ApoJ (a.k.a. clusterin) has been reported to opsonise apoptotic cells (122) and cellular debris (142) by binding histones on cells and phagocytic receptors LRP1 and LRP2 (megalin) on phagocytes (142). ApoH (a.k.a. β2 glycoprotein 1) is recruited to the cell-surface of apoptotic cells (222), and can enhance phagocytosis of apoptotic thymocytes, lipid-symmetric red blood cells and platelet micro-vesicles by macrophages (70, 143). ApoH can associate with phosphatidylserine, which may be sufficient to mediate its opsonic function.

***Calreticulin*** is an eat-me signal for cells undergoing apoptosis or ER-stress, inducing phagocytosis by activating the phagocytic LRP1 receptor (58, 59, 114, 115). However, calreticulin is a soluble protein that can be released from cells extracellularly, to bind the surface of cells and function as an opsonin. Calreticulin can bind phosphatidylserine (56), and Wijeyesakere et al. (71) showed that calreticulin directly bound phosphatidylserine exposed on apoptotic cells *via* its C-terminal acidic region, and enhanced phagocytosis of apoptotic fibroblasts by peritoneal macrophages, which was dependent on this phosphatidylserine-binding region. Feng et al. (47) found that calreticulin was actively secreted by macrophages, and bound to and opsonised co-incubated neutrophils, and blocking calreticulin on the neutrophils reduced their phagocytosis by macrophages. Calreticulin bound to the neutrophils *via* galactose (Galβ1➔4GlcNAc) residues of asialoglycans normally hidden by terminal sialic acid residues, so calreticulin acted as an opsonin for desialylated cells (47). Analogously, we found that calreticulin is released by activated microglia and binds to bacteria (via sugars), and induces microglial phagocytosis of the bacteria *via* LRP1 (133). To add to the complexity, calreticulin can also function as a phagocytic co-receptor - for example during the phagocytosis of apoptotic T cells by macrophages, *via* complex formation with macrophage-exposed LRP1 and surfactant proteins SP-A and SP-D (151) and/or C1q (148).

***Cellular Communication Network factor 1 (CCN1, CYR61)*** is an extracellular matrix protein expressed in placental, skeletal, nervous and cardiovascular cells (223). Jun et al. (72) found that CCN1 opsonised apoptotic neutrophils for phagocytosis, by binding phosphatidylserine on the neutrophils and the integrins α_v_β_3_ and α_v_β_5_ on macrophages (72).

***sCD93 (soluble CD93)*** is derived from the membrane protein CD93, and is released from myeloid cells in a soluble form detectable in human plasma (224). sCD93 can opsonise beads and apoptotic cells for phagocytosis by macrophages, *via* the phagocytic receptor CR4 (integrin α_x_β_2_) (146), and CD93^-/-^ mice exhibit impaired phagocytic clearance of apoptotic cells (225). What sCD93 binds to on apoptotic cells is not known, but is mediated by the lectin domain of sCD93, so may be glycans (146).

***Collectins: Mannose-Binding Lectin (MBL), Surfactant Proteins A (SP-A) and Surfactant Protein D (SP-D)***. Collectins are a family of sugar-binding proteins, including MBL, SP-A and SP-D (191, 226). MBL can bind the surface of apoptotic cells along with C1q, which together forms a complex with calreticulin and LRP1 (CD91) present on the surface of local phagocytes to induce engulfment of the apoptotic cell (148). Apoptotic cells shown to be opsonised by MBL include erythrocytes (148), T cells (148) and adipocytes (192). Necrotic cells are also opsonised by MBL (191). MBL can bind phosphatidylserine (84) and asialoglycans (N‐acetyl‐glucosamine, mannose or fucose residues) (226), which may mediate binding to apoptotic cells. MBL can also bind MASP (mannan-binding lectin serine protease) to initiate the lectin-pathway of complement activation, thereby inducing opsonins C3b, iC3b and C4b (191). SP-A and SP-D also act as opsonins for macrophage phagocytosis of several apoptotic cell-types, mediated *via* calreticulin and LRP1 on the macrophage surface (151, 211). Binding of SP-A to apoptotic cells was *via* binding to exposed phosphatidylserine (85). Note, however, that SP-A and SP-D have also been reported to inhibit phagocytosis by activating SIRPα (227).

***Complement proteins C1q, C3b and C4b and cleavage products iC3b, C3c, C3d and C3dg*** can opsonise targets *via* complement receptors CR1, CR2, CR3, CR4 and CRIg (83, 158, 160) expressed by several phagocytes, notably neutrophil monocytes and macrophages (228, 229). C1q can also induce phagocytosis *via* LRP1 (55, 117) or the calreticulin/LRP1 receptor complex (148). C1q, C3b, iC3b and C4b have together been implicated in the opsonisation of a wide range of apoptotic cells, as well as necrotic cells (**Table 3**). C1q and C3b can each bind phosphatidylserine (83, 147), which may mediate their opsonisation of phosphatidylserine-exposing apoptotic cells. However, C1q may also opsonise by binding calreticulin (151), a known C1q receptor and established eat-me signal present on the surface of apoptotic cells. C1q can also bind DNA (230), initiating complement activation, and nucleic acid exposure by apoptotic cells increases detectable C1q and C3b on the apoptotic cell surface (120). Complement can also opsonise neuronal synapses for phagocytosis by microglia (155). Desialylation of neurons caused C1q to bind to neurons, resulting in CR3-mediated microglial phagocytosis of neurites (presumably by inducing C3b deposition) (48). It is unclear why C1q binds more to desialylated neurons, though this may occur *via* binding exposed galactose and N-acetyl-glucosamine residues (113). Desialylation of pentraxin-3 has also been shown to induce C1q binding and complement activation (231). Complement opsonisation of myelin has also been reported (159). C1q binding to a cell can induce the complement cascade resulting in local deposition of C4b and C3b on the cell. C3b and C4b covalently attach to cell surface hydroxyl groups, typically on sugars, opsonising such surfaces for phagocytosis (157, 162). Cell surface sialylation inhibits C3b production and stability *via* recruiting the complement inhibitor Factor H (232). Thus, the complement system of proteins contains multiple opsonins with complex regulation of phagocytosis.

***Developmental endothelial locus-1 (Del-1)*** is a cell-secreted glycoprotein, recently shown to opsonise apoptotic neutrophils for phagocytosis by macrophages (171). Del-1 can bind directly to phosphatidylserine *via* it’s C-terminal domain, and Del-1-mediated phagocytosis required the α_v_β_3_ integrin receptor (171).

***Ficolins*** are group of pattern recognition proteins known to act as opsonins for pathogens *via* binding surface sugar residues such as N-acetyl-glucosamine, but may also opsonise necrotic host cells *via* binding DNA (119) or pentraxins (176).

***Fibronectin*** is a large (440 kDa) soluble glycoprotein found at high levels in plasma (233), and early reports indicated that fibronectin could enhance monocyte phagocytosis of erythrocytes (172), possibly *via* Fc receptors expressed by the monocyte. Subsequently, fibronectin was reported to bind and opsonise apoptotic Jurkat T cells and activate macrophage phagocytosis of such cells *via* also binding and activating the α_5_β_1_ integrin on the macrophages (174).

***Galectin-3*** is a β-galactoside-binding protein expressed in myeloid cells including macrophages, monocytes, dendritic cells and neutrophils (234). Galectin-3 is released from inflamed macrophages, and can bind to exposed galactose residues on cells to opsonise these cells (49). Galectin can enhance the uptake of apoptotic neutrophils by monocyte-derived macrophages, blocked by lactose, which competes for sugar binding (178). Whilst it usually exists as a monomer, binding to galactose residues induces oligomerisation, which may facilitate the bridging between phagocytes and target cells during phagocytosis (235). Galectin-3 can directly bind the phagocytic receptor MerTK, and stimulate macrophage phagocytosis of apoptotic T cells (177). We found that galectin-3 binds to desialylated cells and induces microglial phagocytosis of cellular debris and neuroendocrine cells, inhibited by blocking MerTK (49). More recently, we have shown that galectin-3 can opsonise gram-negative bacterial *E. coli* for phagocytosis by microglia *via* MerTK (133). Thus, galectin-3 can opsonise both host and foreign targets for phagocytic elimination in mammalian systems.

***Growth arrest-specific protein 6 (Gas6) ***is an extracellular protein ligand for the phagocytic receptor MerTK (as well as for other TAM members Tyro3 and Axl) (75, 179, 180). Gas6 binds phosphatidylserine, which mediates the phagocytosis of apoptotic thymocytes by macrophages (74), and Gas6-dependent phagocytosis is abolished by genetic deletion of MerTK (180). Gas6 has further been described to induce phagocytosis of apoptotic cells by microglia (181), and also of photoreceptor outer segments by retinal pigment epithelial cells (182).

***High-molecular weight kininogen (HK)*** is a serum protein, increased by inflammation, found to opsonise apoptotic cells by binding phosphatidylserine on these cells and activating the urokinase plasminogen activator receptor (uPAR) on macrophages (183).

***Milk fat globule-epidermal growth factor 8 (MFG-E8)*** is an extracellular protein ligand for the integrin receptors α_v_β_3_ and α_v_β_5_ (236). MFG-E8 is secreted from dendritic cells (237) and macrophages (197), and can opsonise apoptotic cells for phagocytic removal (236). Soluble MFG-E8 binds phosphatidylserine exposed on the target cell (77) and either of the integrin receptors α_v_β_3_ (76) or α_v_β_5_ (193) on the phagocyte, thus inducing phagocytosis. MFG-E8 can promote phagocytosis of defective red blood cells (sickle cells) (196), apoptotic lymphocytes (195) and live neurons (198) by macrophages, photoreceptor cells or cell segments by retinal pigment epithelia (193), and apoptotic thymocytes by fibroblasts expressing α_v_β_3_ (76).

***Pentraxins: C-reactive protein, serum amyloid P and PTX3***. Pentraxins (PTX) are extracellular pentameric proteins that function as pattern recognition molecules (238). Three pentraxins - C-reactive protein (CRP, a.k.a PTX1), serum amyloid P (SAP, a.k.a PTX2) and PTX3 opsonise mammalian and microbial cells for phagocytosis. CRP binds and opsonises apoptotic T cells for phagocytosis by macrophages (169, 170), and also erythrocytes for phagocytosis by peripheral blood mononuclear cells (PBMCs) (168). In both cases, opsonisation was mediated by phagocytic Fc receptors. SAP also binds and opsonises apoptotic cells, including T cells and neutrophils (170), for phagocytosis by macrophages, again mediated by phagocytic Fc receptors. As noted previously, PTX3 has been described as an eat-me signal. However, Ma et al. (176) showed that exogenous PTX3 associates with apoptotic or necrotic (but not viable) T cells, where it complexes with ficolin-1 to enhance phagocytosis of these cells by macrophages. Lech et al. (239) found that murine macrophages lacking PTX-3 had a reduced ability to phagocytose apoptotic cells with normal PTX-3 levels, confirming the importance of non-target-cell PTX-3 for phagocytosis. It is unclear to what CRP, SAP and PTX-3 bind to on target cells, although each can bind to C1q (167, 205), which might mediate the binding to apoptotic cells (169).

***Properdin*** is a soluble protein released by leukocytes, and can modulate inflammation by stimulating the alternative pathway of complement activation (240). Properdin can bind exposed DNA and induce deposition of the opsonin iC3b on apoptotic cells, indirectly stimulating phagocytosis *via* complement (118). However, properdin can opsonise independently of complement by binding sulphated glycosaminoglycans exposed on apoptotic and malignant T cells, inducing their phagocytosis by macrophages or dendritic cells *via* unknown receptors (199).

***Protein S (Pros1)*** is a serum protein that can bind phosphatidylserine (81) and the phagocytic receptor MerTK (241). McColl et al. (200) found that the phagocytosis of apoptotic neutrophils by dexamethasone-treated macrophages involved opsonisation by protein S signalling *via* MerTK. Similarly, phagocytosis of photoreceptor cells by retinal pigment epithelia was mediated by Pros1 and MerTK (75). Protein S binding to phosphatidylserine opsonises apoptotic lymphocytes for phagocytosis by macrophages (80). Opsonisation by protein S has also been demonstrated in macrophage phagocytosis of apoptotic T cells (202) and apoptotic neutrophils (203). Protein S can also activate the phagocytic receptor Tyro3 (75), but it is unclear where this might regulate phagocytosis.

***Thrombospondin (TSP-1)*** is an extracellular matrix protein expressed by endothelial cells, monocytes and macrophages (242). Extracellular secretion of TSP-1 by cells increases during apoptosis (86), and extracellular TSP-1 has been shown to opsonise apoptotic neutrophils (213), eosinophils (214) and fibroblasts (86) for phagocytosis by macrophages. TSP-1 can bind phosphatidylserine (212), which may mediate opsonisation by TSP-1. However, Moodley et al. (86) found that TSP-1 acted as a bridging protein between CD36 on apoptotic fibroblasts and CD36 on macrophages, and induced phagocytosis independent of phosphatidylserine. TSP-1 also indirectly associates with α_v_β_3_ *via* the integrin-associated protein (IAP) (243), which may also mediate TSP-1 opsonisation in contexts of efferocytosis.

***Tubby (TUB) and tubby-like protein 1 (TULP1)*** - two structurally related members of the tubby protein family – are expressed in the brain (244) and reside intracellularly, but can also be secreted by cells (245). Extracellular TUB and TULP1 enhance the phagocytosis of photoreceptor outer segments by retinal pigment epithelia by activating the phagocytic receptor MerTK (215, 216). Moreover, TULP1 enhanced the microglial phagocytosis of apoptotic (but not healthy) T cells, and also of neuroblastoma-derived membrane vesicles, both mediated *via* MerTK (218). TULP1 can also interact with the other TAM receptors Tyro3 and Axl (215). It is unclear to what TUB binds to on target cells, although TULP1 can bind phosphatidylserine (217), which likely mediates its opsonic effect.

### Don’t-Eat-Me Signals

**Figure 6:**
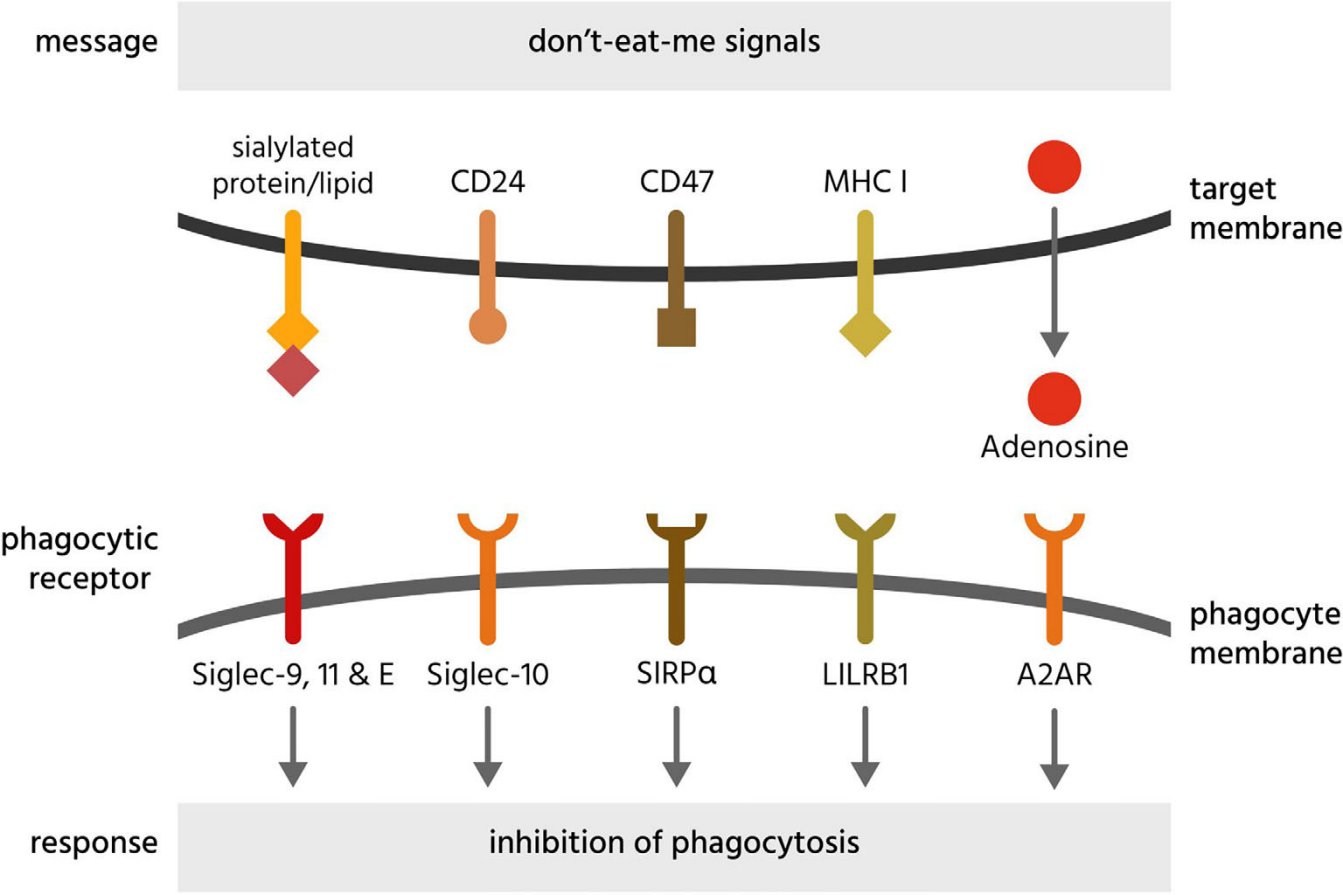
Don’t-eat-me signals and their receptors. Don’t-eat-me signals are molecules exposed on or released from a target cell to directly inhibit phagocytosis of that cell by a phagocyte. The figure illustrates eat-me signals on or from mammalian target cells. A2AR, adenosine 2A receptors; CD, cluster of differentiation; LILRB1, leukocyte immunoglobulin-like receptor subfamily B member 1; MHC I, major histocompatibility complex 1; Siglec, sialic acid-binding immunoglobulin-type lectin; SIRPα, signal regulatory protein α.

The vast majority of cells in the body express don’t-eat-me signals in order to prevent themselves being eaten by phagocytes. Don’t-eat-me signals are signals on or from target cells that inhibit the phagocytosis of these cell. **Table 4** lists known don’t-eat-me signals, whilst **Figure 6** illustrates don’t-eat-me signals and their binding partners.

**Table 4:** Don’t-eat-me signals.

Name	Phagocytic receptor(s)	Phagocytic function(s)
**Adenosine**	A2AR (246)	Macrophage phagocytosis of lymphoma cells (246)
**CD24**	Siglec-10 (247)	Macrophage phagocytosis of cancer cells (247)
**CD47**	SIRPα (248, 249)	Macrophage phagocytosis of erythrocytes (250–252), apoptotic epithelial cells (249), and cancer cells (253)
**MHC I (β2-microglobulin)**	LILRB1 (254)	Macrophage phagocytosis of cancer cells (254)
**Sialic acid residues**	Siglec-9 (255), -11 (256) & -E (257)	Monocyte phagocytosis of apoptotic lymphocytes (54) and senescent erythrocytes (258)

***Adenosine*** can be released by stressed or dying cells and inhibits phagocytosis of such cells *via* adenosine 2A receptors (A2AR) on phagocytes (246). Antibody-induced phagocytosis of cancer cells was inhibited by adenosine and enhanced by knockout or inhibition of A2AR receptors *in vivo* (246).

***CD24*** is a sialylated glycoprotein present on the surface of B cells and neutrophils, inhibiting the phagocytosis of such cells by binding and activating Siglec-10 on phagocytes (247). CD24 is upregulated on ovarian or breast cancer cells, and Barkal et al. (247) found that blocking both CD47 and CD24 synergistically enhanced phagocytosis, indicating redundancy of these two membrane-bound don’t-eat-me signals. Siglec-10 binding to CD24 is partly mediated by sialic acid residues on CD24 but also some amino acid residues (247), indicating a level of specificity towards CD24.

***CD31 (PECAM-1)*** is expressed on the surface of leukocytes, macrophages and endothelial cells, and was reported to promote the detachment of leukocytes from macrophages under flow (259). During apoptosis, CD31 can change conformation to promote attachment and enable phagocytosis (259), possibly by stimulating the α_5_β_1_ integrin receptor (174). However, this role of CD31 has not confirmed, and the reported mechanism does not conform to a traditional don’t-eat-me signal, but rather a detachment signal under flow.

***CD47*** is a transmembrane receptor expressed by virtually all cells, and inhibits phagocytosis of cells by binding and activating the transmembrane receptor SIRPα on phagocytes (249, 250). Loss of CD47 expression induces phagocytosis of apoptotic cells (249) and senescent erythrocytes (250). Expression of CD47 appears sufficient to inhibit phagocytosis mediated by antibodies and complement (251). Loss of CD47 or antibody blockade is sufficient to induce phagocytosis of some cells (erythrocytes and cancer cells) (252, 253), but not other cells (260), suggesting either that other don’t-eat-me signals can replace CD47 in particular cells, or that CD47 blockade is sufficient to induce phagocytosis only in cells exposing eat-me signals. CD47 can be cleaved by metalloproteases on apoptotic cells, to remove this don’t-eat-me signals but also release the soluble ectodomain of CD47 (sCD47), which can antagonise SIRPα on phagocytes to stimulate phagocytosis. Thus, sCD47 may potentially act as a soluble eat-me signal (261).

***CD200 (OX-2 membrane glycoprotein)*** is a transmembrane protein that is typically expressed on the surface of hematopoietic-derived cells, as well as B-cells, activated T cells, endothelial cells and neuronal cells (262). Expression of CD200 on endothelial cells was found to inhibit phagocytosis of those cells by macrophages, possibly by engaging the CD200 receptor, CD200R (263). However, CD200 inhibits inflammation *via* CD200R (262), so it is possible that the inhibition of phagocytosis is indirect *via* inhibiting inflammation.

***MHC class I (major histocompatibility complex class I; MHC1)*** is a transmembrane protein complex present on the surface of all host cells (except erythrocytes) and is a dimer of a variable α-subunit and an invariant β2-microglobulin. This β2-microglobulin subunit of MHC1 inhibits phagocytosis by binding and activating LILRB1 (leukocyte immunoglobulin-like receptor subfamily B member 1) on macrophages (254). Because MHC1 is also a core component of adaptive immunity, it is downregulated on many cancer cells and virally-infected cells, potentially making them susceptible to phagocytosis by macrophages (254).

***Programmed death-ligand 1 (PD-L1)*** is transmembrane protein, expressed on immune cells, epithelial cells, and vascular endothelial cells, suppressing adaptive immunity by activating the inhibitory receptor programmed cell death protein 1 (PD-1) on T cells. Recently, it was reported that PD-L1 on tumour cells acted as a don’t-eat-me signal to inhibit their phagocytosis by tumour associated macrophages by activating PD-1 on the macrophages (264). However, this paper showed little evidence that PD-L1 directly inhibited phagocytosis, rather than inhibiting inflammatory activation of the macrophages, so PD-L1 status as a don’t-eat-me signal requires verification.

***Sialic acid (N-acetylneuraminic acid)*** residues on the surface of virtually all cells inhibits phagocytosis of such cells by engaging and activating sialic acid-binding immunoglobulin-type lectin receptors (Siglec-9, -10, -11 and -E) on phagocytes (265). Sialic acid residues terminate the sugar chains of most glycoproteins and glycolipids on the cell surface, but these residues can be removed by sialidases (266). Loss of sialic acid residues (known as desialylation) can induce phagocytosis of apoptotic cells (54) and senescent cells (258). Cancer cells are hypersialylated and may overexpress highly sialylated mucins to prevent themselves being phagocytosed (267).

### Negative Opsonins and Phagocyte Suppressants

**Figure 7:**
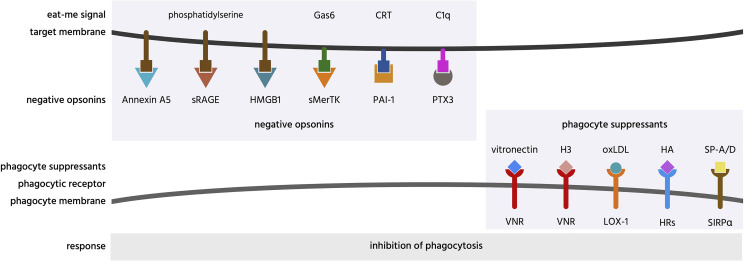
Negative opsonins and phagocyte suppressants, and their potential mechanisms of action. Negative opsonins are extracellular proteins that, when bound to a target cell, inhibit phagocytosis of that cell by phagocytes; a phagocyte suppressant is a normally soluble, extracellular molecule, which, when bound to a phagocyte discourages it from phagocytosing targets. CRT, calreticulin; H3, histone H3; HA, hyaluronic acid; HMGB1, high mobility group box 1 protein; HRs, hyaluronic acid receptors; LOX-1, lectin-like OxLDL receptor 1; oxLDL, oxidised low-density lipoprotein; PAI-1, plasminogen activator inhibitor-1; PTX3, pentraxin 3; SIRPα, signal regulatory protein α; sMerTK, soluble Mer tyrosine kinase; sRAGE, soluble receptor for advanced glycation end products; SP, surfactant protein; VNR, vitronectin receptors.

Some extracellular proteins, not derived from the target cell, can inhibit phagocytosis, and we refer to these as either ‘negative opsonins’ (‘nopsonins’ for short) if they bind the target cell, and ‘phagocyte suppressants’ if they bind the phagocyte. Nopsonins and phagocyte suppressants are summarised in **Table 5** and depicted in **Figure 7**. Note, however, it has rarely been demonstrated that these proteins can inhibit phagocytosis physiologically *in vivo*. Why might there be negative regulators of phagocytosis? Opsonins are probably expressed to amplify phagocytosis of eat-me signalling cells in particular conditions, such as inflammation, or alternatively to direct phagocytosis *via* particular phagocytes or phagocytic receptors, which determines what happens to the target cell. So, reasons for having negative regulators of phagocytosis might include: to suppress (or set a threshold for) phagocytosis of eat-me signalling cells in other conditions, such as in the healthy adult, or alternatively to suppress phagocytosis *via* particular phagocytes or particular receptors, thereby directing the fate of target cells.

**Table 5:** Negative opsonins and phagocyte suppressants.

Name	Mechanism of action	Phagocytic function(s)
**Annexin A5 (AnxA5)**	Potential negative opsonin by blocking phosphatidylserine on target (268)	Microglial phagocytosis of apoptotic glioma cells (269); macrophage phagocytosis of apoptotic lymphocytes (129); smooth muscle cell phagocytosis of apoptotic smooth muscle cells (268)
**High mobility group box 1 (HMGB1)**	Potential negative opsonin by blocking phosphatidylserine (270); potential phagocyte suppressant by blocking α_v_β_3_ (271)	Macrophage phagocytosis of apoptotic neutrophils and thymocytes (270, 271)
**Hyaluronic acid**	Potential phagocyte suppressant by coating phagocyte with negative charge (267)	Macrophage phagocytosis of apoptotic cells (267)
**Oxidized low-density lipoprotein (OxLDL)**	Potential phagocyte suppressant by blocking LOX-1 on phagocytes (63)	Endothelial cell phagocytosis of aged erythrocytes and apoptotic cells (63)
**Pentraxin-3 (PTX3)**	Potential negative opsonin, possibly by blocking C1q on target (272)	Dendritic cell phagocytosis of apoptotic HeLa cells (273) and T cells (274); macrophage phagocytosis of apoptotic polymorphonuclear leukocytes (275); microglial phagocytosis of apoptotic thymocytes (276); macrophage phagocytosis of neurons (277)
**Plasminogen activator inhibitor-1 (PAI-1)**	Potential negative opsonin, possibly by blocking calreticulin on target (278)	Macrophage phagocytosis of viable and apoptotic neutrophils (278)
**Soluble Mer tyrosine kinase (sMerTK)**	Negative opsonin by blocking Gas6 and protein S on target (279, 280)	Macrophage phagocytosis of apoptotic cells. Retinal pigment epithelial cell phagocytosis of photoreceptor outer segments (280).
**Soluble receptor for advanced glycation end products (sRAGE)**	Negative opsonin by blocking phosphatidylserine on target (94)	Alveolar macrophage phagocytosis of apoptotic thymocytes (94)
**Soluble urokinase-type plasminogen activator receptor (suPAR)**	Possibly binding uPAR on apoptotic cells or αvβ3 on phagocyte (281)	Macrophage phagocytosis of viable and apoptotic neutrophils (281)
**Surfactant proteins A (SP-A) and D (SP-D)**	Possible phagocyte suppressant by activating SIRPα on phagocyte (234)	Macrophage phagocytosis of apoptotic cells (227)
**Vitronectin**	Possible negative opsonin by blocking uPAR on target (281), possible suppressant by blocking αvβ3 and αvβ5 on phagocyte (282)	Macrophage phagocytosis of apoptotic thymocytes and neutrophils (282); CS-1 phagocytosis of CEM-1 and T cells (283)

***Annexin A5 (AnxA5)*** is normally an intracellular protein, but is present in serum, and can inhibit phagocytosis of apoptotic cells by binding and blocking phosphatidylserine exposed on the apoptotic cell surface (129, 268, 269, 284). Thus, annexin A5 is a potential negative opsonin.

***High mobility group box 1 protein (HMGB1) ***is normally located in the nucleus, but can be released extracellularly during inflammation, and elevated levels are present in blood plasma in inflammatory states (270). HMGB1 can inhibit phagocytosis of apoptotic neutrophils by binding and blocking phosphatidylserine on neutrophils (270), and/or binding and blocking the phagocytic receptor α_v_β_3_ on phagocytes (72, 271). Thus, HMGB1 is potentially both a negative opsonin and a phagocyte suppressant.

***Histones ***are normally located in the nucleus, but can be released onto the surface of the cell to act as eat-me signals (see above), or released extracellularly during inflammation (285). Histone H3 was reported to inhibit macrophage phagocytosis of apoptotic neutrophils and thymocytes, by blocking the phagocytic receptors MerTK and the integrin receptor α_v_β_5_ (286). However, this has not been verified at physiologically relevant levels. Thus, histone H3 is a potential phagocyte suppressant.

***Hyaluronic acid (HA, hyaluronan)*** is a glycosaminoglycan component of extracellular matrix that can bind some cells including macrophages coating them in a negative charge that can inhibit macrophage phagocytosis of other cells (267). Thus, OxLDL is a potential phagocyte suppressant.

***Oxidized low-density lipoprotein (OxLDL)*** is LDL with oxidised lipids, and is found in serum, and may contribute to atherosclerosis. OxLDL is reported to inhibit endothelial cell phagocytosis of aged and apoptotic cells *via* blocking LOX-1 on endothelial cells (63). Thus, OxLDL may be a phagocyte suppressant.

***Pentraxin 3 (PTX3)*** is a soluble protein found in plasma and released by inflammatory-activated cells (287). PTX3 strongly binds to complement factor C1q and enhances C1-mediated deposition of C3 on cells, thereby enhancing phagocytosis (272). As mentioned previously, PTX3 can act as an opsonin for apoptotic cells (67, 68), but it is unclear whether this is in fact indirect *via* binding to C1q. PTX3 has also been found to inhibit phagocytosis of apoptotic cells in certain contexts (273–277), but again the mechanisms are unclear, possibly again by binding C1q (272). Thus, PTX3 may also act as a negative opsonin.

***Plasminogen activator inhibitor-1 (PAI-1)*** is a serum protein reported to inhibit phagocytosis of viable and apoptotic neutrophils by macrophages, possibly by interfering with calreticulin on the surface of neutrophils (278). Macrophages showed increased phagocytosis of neutrophils from PAI-1 knock-out mice, which was later reversed in the presence of exogenous PAI-1 (278). PAI-1 also inhibited microglial phagocytosis, apparently *via* vitronectin and Toll-like receptors (288). Thus, PAI-1 is a potential negative opsonin and phagocyte suppressant.

***Soluble intercellular adhesion molecule 5 (sICAM-5)*** is the soluble ectodomain of the transmembrane protein ICAM-5. Activated neurons can release sICAM-5, which binds integrins on microglia to inhibit microglial adhesion, activation and phagocytosis (289). However, supraphysiological levels of soluble ICAM-5 were used to inhibit microglial phagocytosis of beads, and this action might be *via* acting on microglial activation or adhesion, rather than on phagocytosis *per se*. As sICAM-5 is present in plasma it might act as a physiological phagocyte suppressant, but this has not investigated.

***Soluble Mer tyrosine kinase (sMerTK)*** is the soluble ectodomain of the phagocytic receptor MerTK. sMerTK is cleaved from MerTK by metalloproteases, and is present in plasma (261). sMerTK is released by activated macrophages and inhibits macrophage phagocytosis of apoptotic cells by binding opsonins ProteinS or Gas6, preventing their binding to full length MerTK (279). Phagocytosis of photoreceptor outer segments by retinal pigment epithelial (RPE) induces release of sMerTK that inhibits further phagocytosis by binding the opsonins (280). Increasing sMerTK release further inhibited phagocytosis, while inhibiting sMerTK release stimulated phagocytosis (280), indicating that sMerTK is a negative opsonin.

***Soluble receptor for advanced glycation end products (sRAGE)*** is produced by the proteolytic cleavage of the receptor RAGE or by alternative splicing of RAGE mRNA (290). RAGE was shown to act as a macrophage receptor for phosphatidylserine on apoptotic cells, while sRAGE was shown to bind to phosphatidylserine on apoptotic cells, and thereby inhibit phagocytosis of such cells (94) by acting as a negative opsonin. Whether it can do this physiologically is unknown, but sRAGE levels in serum change with inflammation and disease states (290).

***Soluble urokinase-type plasminogen activator receptor (suPAR)*** is present in plasma and released from membrane-bound uPAR following cleavage by phospholipase C or D (291). Park et al. (281) reported increased macrophage phagocytosis of neutrophils if uPAR were knocked out in either the macrophages or the neutrophils, and in both cases addition of suPAR inhibited phagocytosis, but the mechanisms are unclear.

***Surfactant protein A (SP-A) and surfactant protein D (SP-D*)** are collectins and extracellular proteins that can act as opsonins (see *‘Opsonins’* section). However, SP-A and SP-D have also been reported to inhibit phagocytosis (as a phagocyte suppressant) by activating SIRPα on macrophages (227). The authors speculated that these opposite effects are determined by whether SP-A and SP-D bind to phagocytes *via* the N-terminal that engages the calreticulin/CD91 receptor or the C-terminal that engages SIRPα.

***Vitronectin*** is a protein found on the surface of cells and in a soluble form in the extracellular matrix (248). Soluble vitronectin was reported to inhibit macrophage phagocytosis of apoptotic cells *in vitro* and *in vivo*, both by blocking uPAR on apoptotic cells and by blocking the vitronectin receptor on macrophages (282). Thus, vitronectin may be both a negative opsonin and a phagocyte suppressant.

## The Phagocytic Code for Particular Phagocytic Targets

Having introduced the components of the phagocytic code, we will now outline how they operate to regulate the phagocytosis of particular targets below.

### Healthy Cells

As far as we know, healthy self-cells are not normally phagocytosed, unless they are excess to requirements or somehow senescent. Thus, the vast majority of host cells are not phagocytosed, preventing loss of healthy cells, but how is this achieved? We only know the answer to this in specific contexts, so much of what follows is speculative. Firstly, most healthy cells do not expose eat-me signals, such as phosphatidylserine (292). However, activated cells (with increased cytosolic calcium) can reversibly expose phosphatidylserine (293), and the eat-me signal calreticulin is constitutively exposed on neutrophils (294). Thus, there is probably a need for healthy cells to express don’t-eat-me signals, and indeed most healthy host cells express CD47 and MHC1 and are sialylated, inhibiting their clearance by phagocytosis (235, 295, 296). In healthy tissues, opsonin levels are relatively low (because most opsonins are induced by inflammation), and in both healthy and inflamed tissues, opsonins do not normally bind to healthy cells. Thus, healthy, self-cells can avoid phagocytosis in principle by: expressing don’t-eat-me signals, not releasing find-me signals, not exposing eat-me signals, and not binding opsonins. However, we don’t actually know the extent to which healthy cells are phagocytosed as part of physiological turnover of tissues – and it would be useful to know this. Parts of healthy cells are phagocytosed, for example, synapses (see below) and it has been suggested that healthy microglia phagocytose their own processes (297), but we don’t know the extent of cellular self-eating generally. Healthy self-cells may be aberrantly phagocytosed in some pathologies associated with inflammation (see below). For example, activation of macrophages with CpG DNA, interferon-γ, and anti-interleukin-10 receptor antibody results in macrophage phagocytosis of live T cells, B cells and myeloid cells *via* ICAM-1 or VCAM-1-mediated adhesion (298).

### Excess Cells During Development and After Inflammation

A variety of cells, especially during development, become redundant and can be considered ‘excess’ cells, needing removal by phagocytosis. In some cases, the phagocytosis is induced by the cell undergoing apoptosis, but in other cases the excess cells are phagocytosed when alive, which raises the question of what signals mediate this phagocytosis of apparently healthy cells. For example, excess neurons in the retina are phagocytosed alive by microglia during development apparently *via* tagging new-born neurons with C1q and microglial phagocytosis of these *via* the CR3 receptor (299).

Many myeloid cells expand during infection and become redundant and potentially damaging post-infection, so are preferentially phagocytosed, usually as a result of phosphatidylserine exposure on live cells. For example, macrophages can induce phosphatidylserine exposure on live neutrophils, which enables the macrophages to phagocytose these neutrophils if the macrophages also release MGF-E8 to bind the phosphatidylserine and vitronectin receptor (300). Inflammatory-activated neutrophils expose phosphatidylserine and oxidise this to lysophosphatidylserine, which induces phagocytosis of such neutrophils by macrophages (301). However, activated neutrophils also desialylate their surface (302), and macrophages can release calreticulin that binds to desialylated neutrophils to induce macrophage phagocytosis of such neutrophils (47). Antigen recognition by live CD8+ T cells induces phosphatidylserine exposure on these cells (303), and the phosphatidylserine receptor TIM-4 has been shown to mediate phagocytosis of antigen-specific T cells post-infection (304).

### Apoptotic Cells

Apoptosis is cell death mediated by Bcl-2 homologous proteins and/or caspase activation, resulting in phosphatidylserine exposure on an intact plasma membrane, which normally suppresses inflammation (305, 306). Phosphatidylserine on apoptotic cells is then bound by opsonins MFG-E8 or Gas6, which then bind and activate the phagocytic vitronectin receptors and MerTK, respectively (**Table 3**). Other phagocytic receptors bind phosphatidylserine directly, such as Tim4, which acts in conjunction with MerTK to induce phagocytosis of apoptotic cells (**Table 3**). The full range of phagocytic receptors binding to phosphatidylserine on apoptotic cells includes BAI-1, CD300f, CD36, LOX-1, PSR, RAGE, Stablin-1 & -2, TIM-1 & -4 and TREM2 (**Table 2**). The full range of opsonins binding to phosphatidylserine on apoptotic cells includes Annexin A1, β2-GP1, calreticulin, CCN1, Gas6, MFG-E8, protein S, C1q, C3b & iC3b, MBL, SP-A, TSP-1 (**Tables 2** and **3**). Other eat-me signals mediating phagocytosis of apoptotic cells include: calreticulin, oxidised phospholipids, DNA and pentraxin-3 (**Table 2**).

Find-me signals released from apoptotic cells includes proteins (chemokines): fractalkine, monocyte chemoattractant protein 1 (MCP-1), interleukin-8 (IL-8), S19 ribosomal protein dimer (RP S19), endothelial monocyte-activating polypeptide II (EMAPII) and split human tyrosyl-tRNA synthetase (mini TyrRS), lipids: lysophosphatidycholine and sphingosine-1-phosphate (S1P), and nucleotides: ATP, ADP, UTP, UDP (**Table 1**). Don’t-eat-me signals and negative opsonins found to block phagocytosis of apoptotic cells include: CD47, sialic acid, annexin A5, histone H3, HMGB1, PAI-1, PTX3, sRAGE, suPAR and vitronectin (**Tables 4** and **5**). CD31 was reported to act as a don’t-eat-me (or detachment) signal, but switch to an eat-me (or attachment) signal as a result of a conformational change induced by apoptosis (259). Analogously, apoptosis can induce desialylation of the apoptotic cell membrane, thereby removing a don’t-eat-me signal (sialic acid residues) and inducing an eat-me signal (asialoglycans) (111). Apoptosis can also expose the eat-me signal calreticulin, while reducing exposure of the don’t-eat-me signal CD47 (58).

### Necrotic Cells and Cellular Debris

Necrotic cells are defined by rupture of the plasma membrane, causing leakage of cellular content that induces inflammation, and if these necrotic cells are not removed rapidly, break up into cellular debris. Phosphatidylserine exposure occurs on all dead and dying cells and debris, but earlier on apoptotic cells, as they have specific mechanisms to induce this. Phosphatidylserine exposure on necrotic cells normally mediates phagocytosis of these cells (307), however, other phagocytic signals from necrotic cells differ from those coming from apoptotic cells, reviewed in (33) and (308). Find-me signals from necrotic cells include formyl-peptides, nucleotides, complement C3a and C5a (**Table 1**). Opsonins shown to mediate phagocytosis of necrotic cells and debris, include complement C1q, C3b and C4b, antibodies IgG and IgM, MBL, pentraxins CRP, SAP and PTX3, AnxA1 and TSP1 (**Table 3**). Necrosis can occur by different mechanisms, including necroptosis, pyroptosis and ferroptosis, but it is unclear whether phagocytosis of these cells occurs *via* different signals.

The biological remnants of cell death are collectively referred to as cellular debris, and their efficient clearance by phagocytes is thought to be important to avoid chronic inflammation and autoimmunity (33). Phosphatidylserine is exposed on cell debris and is probably the main eat-me signal. However, other myelin lipids such as sulfatide and sphingomyelin may contribute to TREM2-mediated phagocytosis of myelin debris by microglia (309). CD47 on myelin debris can act as a don’t-eat-me signal to block its uptake (310). Schwann cells, which myelinate axons in the peripheral nervous system, upregulate phagocytic receptors Axl and MerTK after nerve damage to clear the resulting myelin debris, possibly *via* phosphatidylserine exposure on the debris (311). Phagocytes can also clear myelin debris *via* the complement receptor CR3 (312) or the scavenger-receptor-AI/II (159). Phagocytic clearance of retinal debris occurs *via* α_V_β_3_ and phosphatidylserine receptor (PSR) binding to phosphatidylserine on the debris (313). However, phagocytic uptake of debris from lysed cells by non-professional phagocytes, including fibroblasts and epithelial cells, occurred *via* the opsonin ApoJ (clusterin) binding histones debris and activating uptake through the phagocytic receptors megalin and LRP (142). Galectin-3 can also act as an opsonin for desialylated neuronal debris, facilitating uptake into microglia *via* the MerTK receptor (49). Microglia may be recruited to debris *via* fractalkine and the fractalkine receptor CX3CR1 (314).

### Senescent and Ageing Cells

Cellular senescence refers to irreversible loss of proliferation or other cellular function with age or stress. Senescent cells accumulate in aged organisms, and may contribute to loss of function with age. However, some cells, such as erythrocytes, senesce rapidly as part of physiological turnover. CD47 undergoes a conformation change on senescent erythrocytes, coupled with reduced CD47 expression, promoting the phagocytosis of erythrocytes by macrophages (250, 315). Senescent erythrocytes also expose phosphatidylserine and desialylated membrane glycoproteins - both of which can enhance phagocytosis (316). Neutrophils senescence is even more rapid (24 hours), and is associated with phosphatidylserine exposure, opsonisation by MFG-E8 and annexin A1, and phagocytosis by activated macrophages in the bone marrow (300). Phagocytosis of senescent neutrophils in the spleen depends on phosphatidylserine-binding opsonin Gas6 and its phagocytic receptor MerTK (317). Calreticulin is exposed by aging neutrophils, promoting phagocytosis *via* the phagocytic receptor LRP1 (47, 71). Senescent platelets appear to be phagocytosed as a result of desialylation exposing galactose residues (52). Cells in the post-partem uterus senesce and are removed by macrophages, but the signals driving this are unknown (318). Similarly, macrophages clear senescent cancer cells by unknown phagocytic signals (319).

In the aged brain of mice, there appears to be excessive microglial phagocytosis of synapses and neurons. This appears to be partly mediated by complement, as C3 knockout mice lost less synapses and neurons in the hippocampus, and had improved learning in memory (320). TREM2 knockout mice also had reduced neuronal loss in the hippocampus and substantia nigra (321), suggesting that excessive microglial phagocytosis of neurons may contribute to aging-induced neuronal loss.

### Cancer Cells

Cancer cells have increased expression of both ‘eat-me’ signals and ‘don’t-eat-me’ signals, though expression varies between cancer cell-types and changes with tumour progression (322). Many human cancer cells have the eat-me signal calreticulin on the surface (possibly due to ER stress, or possibly a tumour suppressor mechanism), but overexpress the don’t-eat-me signal CD47 to prevent host phagocytes from phagocytosing the cancer cells (323). Thus, function blocking antibodies and peptides to CD47 or its receptor SIRPα induce macrophage phagocytosis of live cancer cells in culture and *in vivo*, and are being developed as a potential treatment for multiple cancers (322, 324). However, some tumour cells overexpress stanniocalcin 1 (STC1), which obstructs calreticulin exposure on the cell surface, reducing phagocytosis of the cancer cells (325). Macrophages may also recognise and clear haematopoietic cancer cells *via* SLAMF7 heterodimerising between cancer cells and macrophage, and activating CR3 on the macrophage, resulting in phagocytic clearance of the cancer cell (132). MHC-I expression on cancer cells may act as another ‘don’t-eat-me’ signal when bound by the receptor LILRB1 on phagocytes, as disruption of this interaction increases clearance of tumour cells (254). Antibodies bound to cancer cell antigens can induce phagocytosis of the live cancer cell *via* Fc receptors on phagocytes (185). Cancer cells often have increased levels of cell surface sialylation (326), which potentially inhibits phagocytosis of these cells. This inhibition may be partly mediated by sialylation of CD24 on cancer cells, which inhibits macrophage phagocytosis *via* activating Siglec-10 on macrophages (247).

### Infected Cells

Live mammalian cells infected with virus, bacteria or other intracellular pathogens can release signals inducing phagocytosis of the infected cell by phagocytes, thereby limiting infection of the host organism (327). For example, *E. coli*-infection (which presumably involves infected host-cells) induced nucleotides ATP and UDP release from host cells, which stimulated macrophage phagocytosis and reduced bacterial loads *in vivo* (328, 329), and similarly for live host infected with vesicular stomatitis virus (330). Chemokines that attract phagocytes are released by host cells infected with influenza (331), hepatitis C virus (332), and the bacterium *O. tsutsugamushi* (333). Live HIV-infected cells were shown to externalise phosphatidylserine, which induced macrophages to phagocytose these cells, *via* MerTK, Gas6 and Protein S (334). Infection of mouse brain with adenovirus caused phosphatidylserine exposure on live brain cells, with subsequent phagocytosis by microglia of the infected cells mediated by MerTK (335). Similarly, human cells infected by *Chlamydia* rapidly and reversibly exposed phosphatidylserine, induced macrophages to phagocytose the live, infected cells (336). Mycobacterium tuberculosis and cytomegalovirus infection caused calreticulin exposure on infected cells (337, 338).

Red blood cells infected with parasitic Plasmodium reduced CD47 levels to induce their phagocytic removal (339). Influenza infection induces desialylation of infected cells, which increases phagocytosis of the infected cells (340). Infected cells rapidly cause alternative complement pathway activation at their surface, which enables binding to phagocytes (341). West Nile virus infection of neurons induced complement tagging of the neurons, and complement-mediated phagocytosis of the live, infected neurons or synapses by microglia in culture and *in vivo* (342).

### Synapses

The phagocytosis of synapses is required for brain development and synaptic plasticity, where less active synaptic connections are phagocytosed by neighbouring microglia and astrocytes (155, 343). Synapses can facilitate phagocytosis by the release of the find-me signal fractalkine to recruit microglia through CX3CR1 binding, so CX3CR1 knock out mice have both reduced synaptic pruning and impaired synapse maturation (35). ATP also acts as a find-me signal leading to the migration of microglia to stressed or damaged neurons or synapses *via* P2Y_12_ receptor binding (29), and this may contribute to microglial phagocytosis of synapses during experience-dependent plasticity (344). Active synapses express CD47 to inhibit their phagocytosis, and knock-out of CD47 in neurons or its receptor SIRPα in microglia increases phagocytosis of active synapses by microglia (345). To direct phagocytosis of inactive synapses, complement opsonins C1q and iC3b have been reported to mark synapses for pruning, so knockout of C1q, C3 or CR3 prevents synaptic pruning during development or ageing (346). Candidate eat-me signals for complement-mediated opsonisation of synapses include asialoglycans (53) and phosphatidylserine (82).

Astrocytes also contribute to pruning *via* the phagocytic receptors Megf10 and MerTK, all being able to bind to, either directly or indirectly, to C1q (343). The opsonin ApoE can also affect astrocyte phagocytosis of synapses, depending on the isoform (141). The microglial phagocytic receptor TREM2 mediates synaptic pruning, as TREM knockout mice have reduced microglial internalisation of synapses and increased synaptic density (347). Excessive microglial phagocytosis of synapses may contribute to neurodegeneration (348, 349), emphasising the clinical importance for better understanding the phagocytic code regulating synapse removal.

## Consequences of Phagocytosis *via* Different Signals: Reprogramming, Killing, Inflammation and/or Antigen Presentation

What are the consequences for the phagocyte following phagocytosis mediated by different phagocytic signals? The phagocyte needs to decide what to do with what has been phagocytosed, and this decision is influenced by the signals mediating the phagocytosis. An immediate question the phagocyte needs to address is whether to try to kill what is in the phagosome - if it has a live pathogen, then it needs to kill it rapidly, although doing so may also damage the phagocyte and surrounding host cells. Phagocytosis mediated by CR3, Fc receptors and TREM2 activates the NADPH oxidase to generate superoxide, hydrogen peroxide and derivative species in the phagosome, which kill enclosed cells and activate proteases (350). The hydrogen peroxide produced can also activate the phagocyte, promoting inflammation (351). If the phagosome contains a pathogen, it may activate pattern recognition receptors present. However, phagocytosis mediated by phosphatidylserine and phosphatidylserine receptors normally inhibits inflammatory activation of the phagocyte and antigen presentation by the phagocyte (352). For example, activation of TAM receptors, such as MerTK, is anti-inflammatory by multiple mechanisms (353), whereas phagocytosis mediated by calreticulin can promote antigen presentation by the phagocyte (354). Phagocytosis of non-apoptotic, live self-cells results in much slower digestion of the phagocytosed cells (298), possibly because lysosomal enzymes can’t get into the live cell. Phagocytosis of cells can also reprogram phagocytes *via* changes in epigenetics, transcription, expression and release of multiple factors (355–357). Most targets are phagocytosed *via* the eat-me signal phosphatidylserine, but the particular opsonin bridging phosphatidylserine to particular phagocytic receptors determines the phagocytes response. For example, Del-1-mediated phagocytosis of phosphatidylserine-exposed neutrophils in inflamed tissue reprogrammes the phagocytosing macrophages into pro-resolution phenotype, for example, secreting anti-inflammatory TGFβ (171).

## Pathologies Involving Phagocytosis and Their Potential Treatment

In various pathologies, phagocytosis plays beneficial roles. During pathogenic infection, phagocytosis of the infectious agent (and of infected cells) *via* innate and adaptive immunity is beneficial, and insufficient phagocytosis can enable disease (3). Thus, upregulating phagocytosis, or opsonising inactivated pathogens with immunogenic opsonins [e.g. calreticulin (133)] that promote both phagocytosis and antigen presentation by dendritic cells, may be therapeutically beneficial. The autoimmune disease lupus may be caused by insufficient phagocytosis of apoptotic cells, resulting in secondary necrosis that promotes presentation of self-antigens and thus autoimmunity (358). In multiple sclerosis, myelin degeneration causes an accumulation of extracellular myelin, and in models of the disease, microglial phagocytosis of myelin debris promotes myelin regeneration through activation of oligodendrocyte precursor cells (359). Following ischemic stroke, microglial phagocytosis can be beneficial in clearing debris and restoring tissue homeostasis (360). In cancer, insufficient phagocytosis of cancer cells due their overexpression of CD47, and animal models benefit from increasing phagocytosis of cancer cells using function blocking antibodies to CD47 or its receptor SIRPα (361). In atherosclerosis, defective phagocytosis of apoptotic cells within atherosclerotic lesions results in inflammation and formation of a necrotic core, which can then drive acute cardiovascular injury (362). Chédiak–Higashi syndrome is also characterised by insufficient phagocytosis (363). For all these cases where phagocytosis helps combats disease, augmenting phagocytosis may be therapeutically beneficial – providing this augmentation does not promote excessive phagocytosis of healthy cell targets, or otherwise promote inflammation, which might itself cause disease.

In other pathologies, phagocytosis plays detrimental roles. In Alzheimer’s disease, excessive phagocytosis of synapses and neurons by microglia may contribute to disease progression, and blocking phagocytosis can ameliorate animal models of the disease (348, 349). Excessive phagocytosis of neurons and/or synapses within the brain has been linked to several other neuropathologies, including Parkinson’s disease, frontotemporal dementia (364), schizophrenia (166), glaucoma (365) and disease resulting from West Nile Virus infection (342). In such cases, inhibiting (rather than augmenting) phagocytosis may be therapeutically beneficial. However, therapies inhibiting detrimental phagocytosis should be designed with the knowledge that inhibiting phagocytosis can cause pathology *per se* (and vice versa for therapies augmenting beneficial phagocytosis).

Other diseases where phagocytosis has been suggested to be dysfunctional include: hemophagocytic lymphohistiocytosis, arthritis, osteoporosis, cystic fibrosis, chronic obstructive pulmonary disease, autoimmune hepatitis, fatty liver disease, primary biliary cholangitis, type 1 diabetes and wound healing (308). For such pathologies, a full understanding of the phagocytic code is important.

## Open Questions on the Logic of the Phagocytic Code

**Figure 8:**
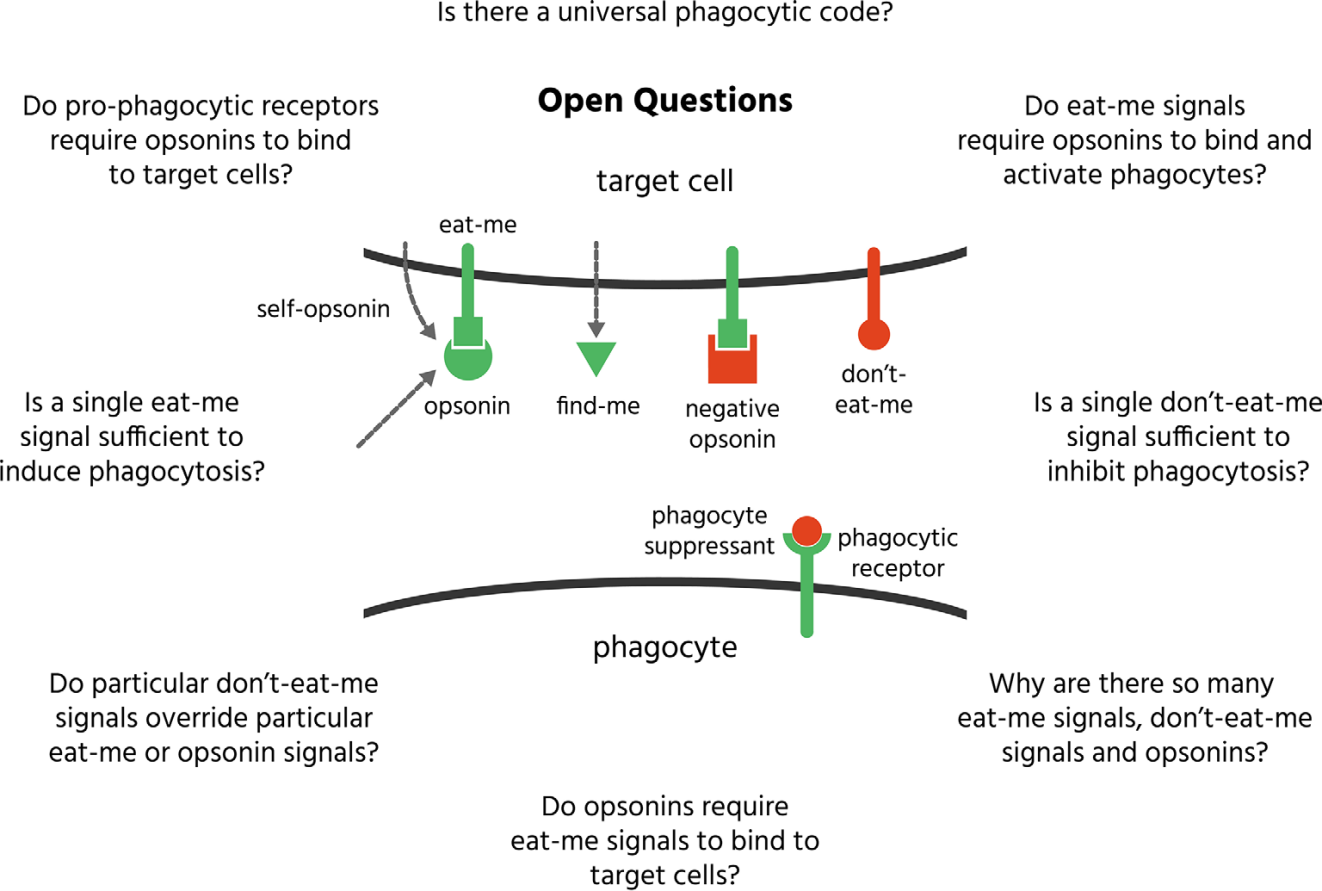
Open questions on the phagocytic code. Multiple signals present on the target cell surface stimulate or inhibit phagocytosis of these targets by phagocytes. How these signals combine to determine whether the target is phagocytosed or not is known as the phagocytic code. However, there are multiple open questions as to how this code works, some of which are listed here and discussed in the text.

When a phagocyte encounters another cell, it may receive several different phagocytic signals encouraging it to phagocytose or not to phagocytose the cell. How does the phagocyte integrate this information and make the crucial decision to eat or not to eat? And is there any simplifying logic to the mass of phagocytic signals? The short answer is that we do not know yet, but below we speculate on some open questions (summarised in **Figure 8**), and make a few tentative generalisations.

### Is a Single Eat-Me Signal Sufficient to Induce Phagocytosis?

Given the range of eat-me signals, is any one sufficient to trigger phagocytosis, or are particular combinations required?

Phosphatidylserine exposure is sufficient to induce phagocytosis of some cells but not others (366). Phosphatidylserine exposure might be insufficient in certain cases due to the presence of don’t-eat-me signals, or the absence of phosphatidylserine-binding opsonins or other co-stimulatory signals. Alternatively, the quantity of phosphatidylserine exposure could be key, i.e. high levels of phosphatidylserine exposure may induce phagocytosis regardless of other don’t-eat-me signals/opsonins present, while low levels may be insufficient without opsonins, additional eat-me signals, or loss of don’t-eat-me signals.

There is some indication that both calreticulin and phosphatidylserine can be required for phagocytosis in some cells and conditions. Gardai et al. (58) found that calreticulin knockout prevented phagocytosis of phosphatidylserine-exposing apoptotic cells, and addition of exogenous calreticulin restored phagocytosis, but this phagocytosis remained phosphatidylserine-dependent. Similarly, we found that microglial phagocytosis of stressed neurons in culture required both calreticulin and phosphatidylserine: calreticulin was constitutively expressed at a low level on cerebellar neurons, and stress induced phosphatidylserine exposure, but blocking either calreticulin or phosphatidylserine prevented phagocytosis (59). The requirement for phosphatidylserine could be overcome by adding high levels of calreticulin, and microglial activation with lipopolysaccharide (LPS) was required for phagocytosis of neurons to occur. Blocking CD47 with anti-CD47 antibodies was not sufficient to induce phagocytosis with these cells, but Gardai et al. (58) found that blocking CD47 was sufficient to induce phagocytosis of viable cells, but this phagocytosis required surface calreticulin. They found that calreticulin was present at low levels on a range of cells, but apoptosis induced calreticulin clumping on the cell surface, together with phosphatidylserine, and away from CD47. Thus, it is possible that clumping of eat-me signals together, and away from don’t-eat-me signals, promotes phagocytosis. However, this hypothesis has not been causally tested.

Is desialylation of cells sufficient to induce phagocytosis? It was reported that desialylation of apoptotic lymphocytes or non-apoptotic lymphoblasts increased their engulfment, whereas desialylation of healthy, resting lymphocytes did not increase their engulfment by monocyte-derived macrophages (54). Thus, desialylation is sufficient to induce phagocytosis of some cells but not others, and can change with conditions. Resting lymphocytes may lack an eat-me signal that is present on apoptotic lymphocytes (e.g. phosphatidylserine) or lymphoblasts (e.g. calreticulin), and the asialoglycans generated by desialylation may be insufficient to induce phagocytosis without asialoglycan opsonins (e.g. calreticulin, C1q, Gal-3 and MBL) being present.

### Is a Single Don’t-Eat-Me Signal Sufficient to Inhibit Phagocytosis?

Is one don’t-eat-me signal sufficient to prevent phagocytosis, making the others redundant? Or is removing one don’t-eat-me signal sufficient to induce phagocytosis? Barkal et al. (254) found that CD47-blocking antibodies induced phagocytosis of many cancer cell lines, while knockout of MHC-I had only a modest effect, however, cell lines resistant to anti-CD47 had high MHC-I expression, and MHC-I knockout substantially increased phagocytosis in these cell line. Similarly, Barkal et al. (247) found that CD24 was expressed in ovarian and breast cancer cells, together with CD47, such that anti-CD24 and anti-CD47 antibodies synergistically induced phagocytosis of these cells by macrophages. Thus, for viable cancer cells, the signals appear to be partially redundant, so that removing more than one don’t-eat-me signal stimulates phagocytosis at least additively, but varying with cell type, depending on their expression of these signals. This emphasises that therapeutic strategies targeting phagocytosis need to consider whether the target is redundant.

### Do Particular Don’t-Eat-Me Signals Override Particular Eat-Me or Opsonin Signals?

There is insufficient evidence to answer this important question for particular eat-me, opsonins and don’t-eat-me signals. However, in order for a specific don’t-eat-me signal to override a specific eat-me signal, the downstream signalling has to interact somewhere before the phagocytic machinery, and the inhibitory signalling must be stronger (and vice versa to induce phagocytosis). An important subset of phagocytic receptors (Fcγ receptors, CR3, TREM2, Dectin-1, CLEC2, MEFG10, Jedi-1) for eat-me signals and opsonins induce phagocytosis *via* ITAM domains and the tyrosine kinase Syk; while most receptors for don’t-eat-me signals (Siglec receptors for sialoglycans and CD24; LILRB1 for MHC-1; SIRPα for CD47) inhibit phagocytosis *via* ITIM domains and tyrosine phosphatases that reverse Syk signalling (367). Thus, don’t-eat-me signals that antagonise Syk signalling may potentially block eat-me and opsonin signalling mediated by the Syk pathway, but not eat-me and opsonin signalling not mediated by Syk, such as that mediated *via* MerTK or α_v_β_3_. This is consistent with the finding that CD47/SIRPα signalling can inhibit phagocytosis mediated by Fcγ receptors and CR3 (282).

### Do Opsonins Require Eat-Me Signals to Bind to Target Cells?

Many opsonins require eat-me signals on the surface of the cell to bind to, but do all of them? There is a rational for opsonin binding to mammalian cells requiring an eat-me signal, because otherwise: how could opsonin binding be specific to target cells rather than healthy cells? The target cell must be displaying something different from healthy cells in order for the opsonin to specifically bind to it, and whatever that difference is, it can be called an eat-me signal. A potential exception is C3b, which appears to bind randomly to cells. However, the specificity of C3b regarding phagocytic regulation arises from: i) the C3 convertase that generates C3b from C3 is specifically located on the target cell by the classical, alternate (C3b) or lectin (MBL) pathway by specific binding to the cell of antibodies and/or C1q, C3b or MBL respectively, and ii) complement factors that specifically bind to target cells and regulate C3b production or disposal, e.g. factor H specifically binds sialylated surfaces and blocks C3b production and induces C3b conversion to iC3b (368). Thus, C3b does not itself bind to an eat-me signal, but it does require an eat-me signal to generate it on the target cell surface and the absence of a don’t-eat-me signal to survive there. Antibody binding to cells does not require a conventional eat-me signal, however, it does require a new epitope, which fits the criteria for an eat-me signal. All other opsonins for mammalian cells either have an identified eat-me signal that they bind to or are presumed to have an as yet unidentified eat-me signal (**Table 3**).

### Do Eat-Me Signals Require Opsonins to Bind and Activate Phagocytes?

Some eat-me signals require opsonins to induce phagocytosis, whilst others don’t, and some eat-me signals can induce phagocytosis in both opsonin-dependent and independent manners (see **Tables 2** and **3**). For example, phosphatidylserine can bind some phagocytic receptors directly, but also can activate phagocytosis indirectly *via* many different opsonins. Similarly, asialoglycans can activate some receptors directly, but is generally recognised *via* multiple opsonins. Calreticulin acts as a self-opsonin (i.e. released from and binding back onto the target cell) that may activate LRP1 directly, but also induces phagocytosis *via* binding the opsonin C1q. Annexin A1 and pentraxin-3 also may act as self-opsonin that activate phagocytic receptors directly.

### Do Pro-Phagocytic Receptors Require Opsonins to Bind to Target Cells?

Most pro-phagocytic receptors on phagocytes bind to target cells *via* opsonins bound to eat-me signals on the target cell (**Tables 2** and **3**). Exceptions are some of the phosphatidylserine receptors, TIM-4, BAI1, PSR and TREM2, which can bind to phosphatidylserine directly (**Table 2**). Note that many opsonins are upregulated in inflammatory states to increase phagocytosis of particular targets.

If most eat-me signals require an opsonin, and most opsonins require an eat-me signal, then an eat-me signal or opsonin alone may not be sufficient to induce phagocytosis. Experimentally, it may not make sense to test whether an eat-me signal or opsonin alone induces phagocytosis. Alternatively, opsonins may be there to amplify the eat-me signal by making it visible or readable by phagocytes, or different opsonins for the same eat-me signal (e.g. phosphatidylserine) may convey different signals to the phagocyte or activate particular types of phagocyte. Opsonins might also potentially act as hub integrating signals by binding several different signals, other opsonins and/or phagocytic receptors to coordinate a response.

### Why Are There so Many Phagocytic Signals?

We now know of over 50 mammalian phagocytic signals. Why so many? Potential explanations for multiple eat-me signals include: i) different eat-me signals can induce different downstream events within the phagocyte once the target cell is phagocytosed, ii) different eat-me signals can induce different phagocytes to phagocytose the cell, and iii) cells are phagocytosed for many different reasons (apoptosis, infection, senescence, etc) that may trigger different eat-me signals to be exposed.

Why are there so many opsonins? Most opsonins work by binding an eat-me signal on the target and a phagocytic receptor on the phagocyte. Thus, because there are several different eat-me signals and phagocytic receptors, multiple opsonins are required to couple them together. Because different eat-me signals can represent different cellular states, and different phagocytic receptors trigger different events within the phagocyte and different fates for the target cells, consequently, different opsonins have somewhat different functions.

### Is There Is a Universal Phagocytic Code?

It is clear that there are somewhat different phagocytic codes for different targets, different phagocytes and different conditions. Unsurprisingly, the phagocytic signals used to remove different phagocytic targets differ, because these target cells are undergoing different cellular processes to identify them as in need of removal. These different targets may also require different treatment by phagocytes, requiring different signals on the target cell (see section on *‘Consequences of Phagocytosis Via Different Signals’*). Different phagocytes may use different phagocytic codes simply as a result of expressing different phagocytic receptors detecting different signals. A rationale for this would be that different phagocytes specialise in the phagocytosis of different targets – for example, neutrophils specialise in phagocytosis of bacteria and therefore express relevant phagocytic receptors to detect such targets. The phagocytic code can change somewhat in different conditions as a result of phagocytes changing the phagocytic receptors they express. For example, during inflammation, different cytokines and inflammatory stimuli can drive the expression of different phagocytic receptors and opsonins (367). And the phagocytosis of targets can itself drive the expression of different phagocytic receptors (369).

If different phagocytes express different receptors (for find-me, eat-me and don’t-eat-me signals and opsonins), and in different conditions, then it follows that all phagocytic signals are conditional – not only on which phagocytic receptors are expressed on the particular phagocyte in a given circumstance, but also on expression levels and activation states of those receptors and their downstream signalling components. However, changes in the phagocytic code for different targets, phagocytes and conditions are limited, and there are commonalities - such as phosphatidylserine and asialoglycans being eat-me signals for most targets.

## Conclusion

It should be clear from the above that we are only just beginning to understand the phagocytic code, its consequences, how it goes wrong in disease and how to manipulate it for treatment. However, seeing the interaction between phagocytes and targets in terms of the phagocytic code helps frame new questions, that will in turn help elucidate this important interaction.

## Author Contributions

Text and tables contained within this review were written and reviewed by TC, JD, AP and GB. Figures were designed by AP, GB, JD and TC, and created by AP. All authors contributed to the article and approved the submitted version.

## Funding

TC received funding from the Biotechnology and Biological Sciences Research Council UK. JD received funding from Eli Lilly and the Biotechnology and Biological Sciences Research Council UK. GB received funding from and from the Medical Research Council UK (No. MR/L010593). This project has received funding from the Innovative Medicines Initiative 2 Joint Undertaking under grant agreement No 115976. This Joint Undertaking receives support from the European Union’s Horizon 2020 research and innovation programme and EFPIA. The authors declare that they have no competing interests.

## Conflict of Interest

The authors declare that the research was conducted in the absence of any commercial or financial relationships that could be construed as a potential conflict of interest.

## GLOSSARY

**Table:**

A2AR	adenosine 2A receptors
ADP	adenosine-5’-diphosphate
AMR	Ashwell-Morell receptor
AnxA1	annexin A1
Apo	apolipoprotein
ATP	adenosine-5’-triphosphate
BAI-1	brain angiogenesis inhibitor 1
Bcl-2	B-cell lymphoma 2
CD	cluster of differentiation
C1q	complement component C1q
C3a	complement component C3a
C5a	complement component C5a
C3aR	C3a receptor
C5aR	C5a receptor (CD88)
CCN1	cellular communication network factor 1
CNS	central nervous system
CR	complement receptor
CRP	c-reactive protein
CX3CL1	chemokine C-X3-C ligand 1 (fractalkine)
CX3CR1	C-X3-C chemokine receptor
CXCR1	C-X-C chemokine receptor type 1
CXCR3	C-X-C chemokine receptor type 3
-1	developmental endothelial locus-1
DNA	deoxyribonucleic acid
EMAP II	endothelial monocyte-activating polypeptide II
ER	endoplasmic reticulum
fMLF	N-formylmethionyl-leucyl-phenylalanine
FPR1	formyl peptide receptor 1
FPR2	formyl peptide receptor 2
G2A	G protein coupled receptor 132 (GPR132)
Gal	galactose
Gal-3	galectin-3
Gas6	growth arrest-specific factor 6
GlcNAc	N-acetylglucosamine
H3	histone 3
HA	hyaluronic acid
HIV	human immunodeficiency virus
HK	high-molecular weight kininogen
HMGB1	high mobility group box 1 protein
IAP	integrin associated protein
ICAM	intercellular adhesion molecule
Ig	immunoglobulin
IL-8	interleukin 8
ITAM	Immunoreceptor tyrosine-based activation motif
ITIM	Immunoreceptor tyrosine-based inhibition motif
LFA-1	lymphocyte function-associated antigen
LILRB1	leukocyte immunoglobulin-like receptor subfamily B member 1
LOX-1	lectin-like OxLDL receptor 1
LPC	lysophosphatidylcholine
LPS	lipopolysaccharide
LRP1	lipoprotein receptor related protein 1
MASP	mannan-binding lectin serine protease
MBL	mannan-binding lectin
MCP-1	monocyte chemoattractant protein 1
MGL	macrophage galactose lectin
MerTK	mer tyrosine kinase
MFG-e8	milk fat globule-epidermal growth factor E8
MHC	major histocompatibility complex
mRNA	messenger RNA
NADPH	nicotinamide adenine dinucleotide phosphate (reduced)
oxLDL	oxidised low-density lipoprotein
oxPL	oxidised phospholipids
P2Y	a family of purinergic receptors
PAI1	plasminogen activator inhibitor-1
PBMC	peripheral blood mononuclear cells
PD-L1	programmed death-ligand 1
PSR	phosphatidylserine receptor
PTX	pentraxin
RAGE	receptor for advanced glycation end products
RAP	receptor associated protein
RP S19	ribosomal protein S19
RPE	retinal pigment epithelia
S1P	sphingosine-1-phosphate
SAP	serum amyloid protein
Siglec	sialic acid-binding immunoglobulin-type lectin
SIRPα	signal regulatory protein α
SLAMF7	signalling lymphocytic activation molecule F7
SP	surfactant protein
STC1	stanniocalcin 1
TAM	tyro, axl, mertk
TIM	T-cell immunoglobulin and mucin
TLR2	toll-like receptor 2
TMEM16f	transmembrane protein 16F
TREM2	triggering receptor expressed by myeloid cells 2
TSP-1	thrombospondin-1
TUB	tubby
TULP1	tubby-like protein 1
UDP	uridine-5’-diphosphate
uPAR	urokinase plasminogen activator receptor
UTP	uridine-5’-triphosphate
VNR	vitronectin receptor
